# An Intrinsically Disordered Region of the Acetyltransferase p300 with Similarity to Prion-Like Domains Plays a Role in Aggregation

**DOI:** 10.1371/journal.pone.0048243

**Published:** 2012-11-01

**Authors:** Alexander Kirilyuk, Mika Shimoji, Jason Catania, Geetaram Sahu, Nagarajan Pattabiraman, Antonio Giordano, Christopher Albanese, Italo Mocchetti, Jeffrey A. Toretsky, Vladimir N. Uversky, Maria Laura Avantaggiati

**Affiliations:** 1 Lombardi Comprehensive Cancer Center, Department of Oncology, Georgetown University, Washington, District of Columbia, United States of America; 2 Department of Neuroscience, Georgetown University Medical Center, Washington, District of Columbia, United States of America; 3 MolBox LLC., Silver Spring, Maryland, United States of America; 4 Sbarro Institute for Cancer Research and Molecular Medicine, Center for Biotechnology, College of Science and Technology, Temple University, Philadelphia, Pennsylvania, United States of America; 5 Department of Molecular Medicine, University of South Florida, Tampa, Florida, United States of America; 6 Institute for Biological Instrumentation, Russian Academy of Sciences, Pushchino, Moscow Region, Russia; National Institute for Medical Research, Medical Research Council, United Kingdom

## Abstract

Several human diseases including neurodegenerative disorders and cancer are associated with abnormal accumulation and aggregation of misfolded proteins. Proteins with high tendency to aggregate include the p53 gene product, TAU and alpha synuclein. The potential toxicity of aberrantly folded proteins is limited *via* their transport into intracellular sub-compartments, the aggresomes, where misfolded proteins are stored or cleared *via* autophagy. We have identified a region of the acetyltransferase p300 that is highly disordered and displays similarities with prion-like domains. We show that this region is encoded as an alternative spliced variant independently of the acetyltransferase domain, and provides an interaction interface for various misfolded proteins, promoting their aggregation. p300 enhances aggregation of TAU and of p53 and is a component of cellular aggregates in both tissue culture cells and in alpha-synuclein positive Lewy bodies of patients affected by Parkinson disease. Down-regulation of p300 impairs aggresome formation and enhances cytotoxicity induced by misfolded protein stress. These data unravel a novel activity of p300, offer new insights into the function of disordered domains and implicate p300 in pathological aggregation that occurs in neurodegeneration and cancer.

## Introduction

Intrinsically disordered proteins (IDPs) are hallmarked by the lack of stable tertiary structure under physiological conditions *in vitro* and *in vivo,* and are increasingly recognized as therapeutic targets [1], [2]. These proteins are very abundant in nature and are proposed to play a physiological role in many biological processes, including transcription and signaling. However, there is also a clear connection between human diseases and protein intrinsic disorder. In the nervous system, several IDPs can acquire stable aberrant conformations becoming aggregation prone and accumulating in the nucleus, cytoplasm or extra-cellular spaces of affected cells, forming distinctive inclusion bodies or fibrillar amyloid [3], [4], [5]. α-Synuclein, TAU and p53 are prototype examples of proteins with an intrinsically disordered conformation that accumulate in neurodegenerative diseases or that are involved in cancer pathogenesis [1], [3], [4], [6].

Cytoplasmic inclusions that resemble pathological aggregates seen in neurodegenerative disorders also form in cultured cells when the proteasome is inhibited. These cytoplasmic organelles are known as aggresomes and exhibit well-defined structure and dynamics [7]. Aggresomes assemble by retrograde transport of misfolded proteins towards the minus end of microtubules, and localize in a specific area of the cell, close to the nucleus and around the microtubule organizing center. This process requires the molecular motor dynein and the deacetylase HDAC6 that act by escorting misfolded poly-ubiquitinated proteins to aggresomes [8], [9]. First identified in the characterization of a mutant form of the cystic fibrosis trans-membrane conducting regulator CFTR-ΔF508 [10], aggresomes also generate a site for replication of various viruses, including Epstein–Barr virus, EBV, and Human Papilloma Virus [11]. Aggresomes form when cells are overwhelmed by misfolded proteins that accumulate due to impairment of proteasomal activity or Endoplasmic Reticulum (ER) stress [9]. Additionally, misfolding and aggregation of many proteins can occur *via* post-translational modifications, alternative splicing, mutations, or oxidative stress [8].

A question that has attracted the attention of many studies pertains to the effects of aggresome formation on cell survival. The original hypothesis of a direct cytotoxicity exerted by protein aggregates has been challenged by several lines of evidence, including the observation that loss of function of the ubiquitin ligase Ube3 in SCA1 mice increases neurodegeneration while decreasing the extent of nuclear aggregates [12]. Similarly, expression of HDAC6, which is necessary for aggresome formation, rescues degeneration caused by proteolytic dysfunction in a *drosophila melanogaster* model of spinobulbar muscular atrophy [13]. These and relayed observations suggest that inclusion bodies might have a cytoprotective effect, acting by either directing misfolded proteins for disruption *via* the autophagic machinery or by simply sequestering them from the cytoplasm thus preventing their toxicity [14]. In this latter instance, the sequestration of misfolded proteins into aggresomes reduces their ability to aberrantly interact with other proteins and removes them from sites of action, in the case of the nervous system, from nerve terminals [9]. Consistent with this protective effect, inhibition of aggresome formation leads to decreased cell viability, and targeting of this process has been proposed as a therapeutic strategy especially in the treatment of cancer [14]. For example, the anti-tumor activity of the proteasome inhibitor, bortezomib, is significantly enhanced when used in combination with HDAC6 inhibitors [9], [15]. However, there is still limited knowledge about the molecular components of aggresomes and their identification might have implications for the understanding of diseases characterized by protein misfolding.

p300 and its homolog *CBP*, play an important role in the execution of multiple biological programs, such as differentiation, senescence and apoptosis [16], [17]. Most, -if not all- of the activities attributed to these proteins take place in the nucleus, *via* regulation of transcription and promotion of acetylation of many factors, including histones. In this study, we identify a novel cytoplasmic activity of p300 unrelated to its acetyltransferase function, and residing in an intrinsically disordered domain with similarities to prion-like domains. Through this region p300 provides an interaction platform for various proteins, including TAU and p53, inducing their aggregation. Our findings unravel a direct role of p300 in aggregation and provide an example of how disordered domains participate in biological processes relevant to human diseases.

## Results

### p300 Localizes in Aggresomes and in Lewy Bodies of Parkinson’s Disease

**Figure 1:**
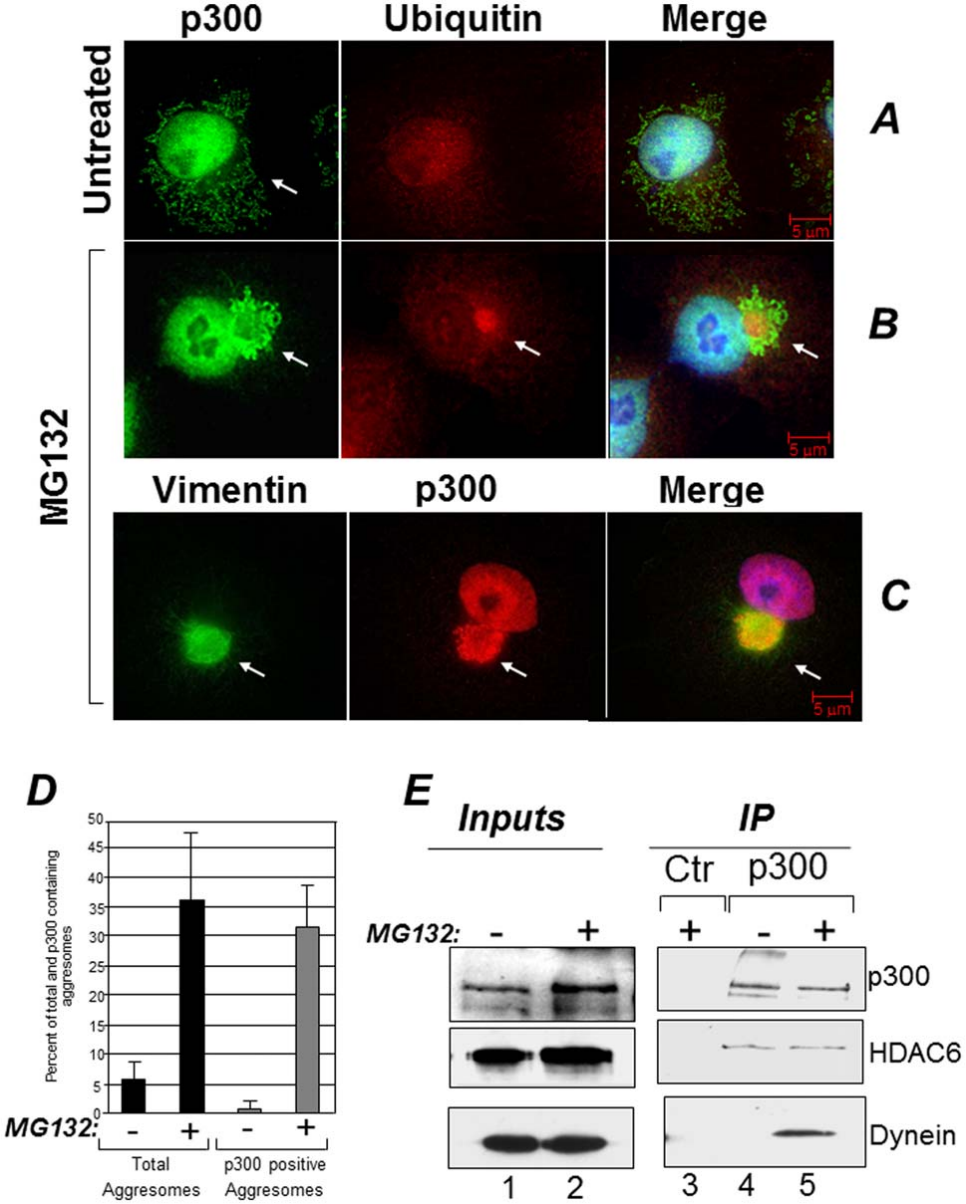
p300 localizes in aggresomes. **A–C**. Cos7 cells were treated with DMSO (A) or treated with 5 µM MG132 for 16 hours (B–C) and cells were stained with a monoclonal mix of anti-ubiquitin (A and B), the anti-p300 polyclonal C20 antibody (A–C), or anti-vimentin monoclonal (C) antibodies (see materials and methods for details on the antibodies). The arrow in panel A, indicates the presence of p300 in diffused cytoplasmic aggregates. The arrows in B and C indicate the position of representative aggresomes, enclosed by vimentin and containing ubiquitin. Cells were subjected to fluorescence deconvolution and image reconstruction by using a Zeiss microscope (Axiovert 200M) with deconvolution capabilities (Axiovision 4.1). The merge panels represent the deconvoluted images from the Alexa-Fluor 488 (green), Alexa-Fluor 568 (red), and DAPI signals. **D.** Quantification of the immuno-fluorescence experiments. The percentage of cells containing aggresomes (black bars) and of aggresomes containing p300 (gray bars) was calculated in mock treated (−) or MG132 treated (+) cells. **E.** Cos7 cells were mock treated (−; lanes 1 and 4) or treated with MG132 (+; lanes 2, 3 and 5) and cell extracts were subjected to immuno-precipitation with a control antibody (lane 3) or with the anti-p300 antibody (lanes 4 and 5). Immuno-precipitation reactions were divided into different aliquots and subjected to immuno-blot with anti p300 (top panel), anti-HDAC6 (mid panel) or anti dynein antibodies (bottom panel), as indicated. Lanes 1 and 2 contains inputs levels of the indicated proteins.

Inhibition of the activity of the proteasome results in accumulation of misfolded ubiquitinated proteins that are segregated into aggresomes [7], [8], [9]. It was previously shown that p300 can associate with ubiquitinated protein species, but it was not clear whether such association occurs in the nucleus or in the cytoplasm [18], [19], [20]. Therefore we studied the localization of p300 and ubiquitin in cells where the global levels of ubiquitinated proteins are increased due to treatment with the proteasome inhibitor MG132. In untreated Cos7 cells, p300 was detectable predominantly in the nucleus, as expected, but also in the cytoplasmic compartment and p300 and ubiquitin did not overtly co-localize (**Figure 1A**). Following MG132 treatment however, a significant fraction of p300 collapsed into aggresomes in a juxta-nuclear position, where it co-localized with both ubiquitin and vimentin (**Figure 1** **,** compare panels B and C with panel A). Indeed, the majority of the aggresomes formed in the presence of MG132 contained p300 (**Figure 1D**). To determine whether this pattern of aggresome localization is cell type specific, we expanded these experiments to other cell lines. p300 was present in aggresomes in the lung cancer cell line H1299 and in human embryonic kidney Hek293 cells (**Figures S1A and S1B**). To then confirm the association with aggresomes detected in immuno-fluorescence, we asked whether p300 forms soluble protein-protein complexes with various known components of these organelles. These experiments showed that p300 interacts with two important constituents of aggresomes, specifically with the cellular motor protein dynein and with HDAC6 (**Figure 1E**) [8]. Interestingly, a similar cytoplasmic pattern of localization could not be clearly seen in the case of CBP, in spite of the high degree of homology between these two proteins (**Figure S2**).

**Figure 2:**
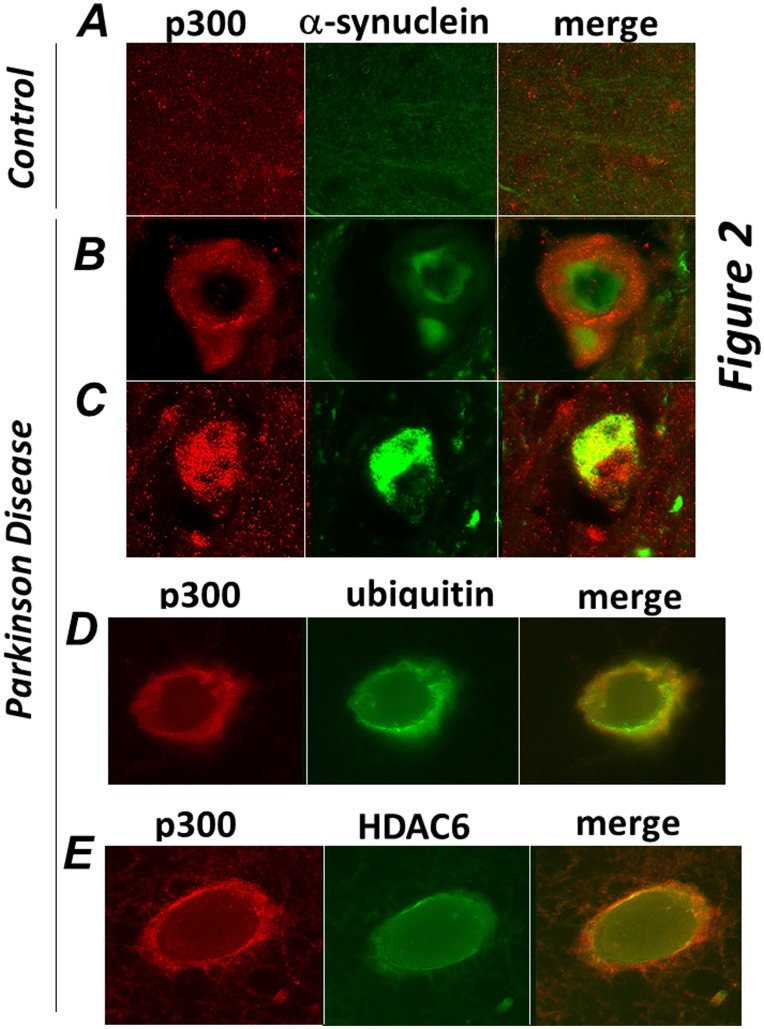
p300 co-localizes with a-synuclein, ubiquitin and HDAC6 in brain of patients affected by Parkinson Disease. Sections of midbrains or cortex from normal control patients (**A**), or from patients by Parkinson Disease (**B–E**) were immuno-stained with the anti-p300 (red) and α-synuclein (green, panels **B,C**), or with anti-ubiquitin (green, panel D), or with anti-HDAC6 (green, panel E) antibodies as indicated at the top of each panel. Different sections from the same patients or from different patients were subjected to analysis.

Aggresomes share similarities with various pathological inclusion bodies in terms of their structure and composition, particularly with Lewy Bodies (LB), which are characteristic of neurodegenerative disorders such as Parkinson Disease (PD) and dementia [8], [21], [22]. Therefore to determine whether p300 can be detected in these types of aggregates we performed immuno-staining of brain sections derived from patients affected by PD or of normal control brains. In the brain of PD patients, Lewy Bodies are identified as large inclusions characterized by the presence of alpha-synuclein, ubiquitin and HDAC6 [8]. While p300 was undetectable in sections derived from normal brains (**Figure 2A**), in the midbrain of PD patients it formed distinct large aggregates that either surrounded (**Figure 2B** **)** or co-localized (**Figure 2C** **)** with pathognomonic a-synuclein positive inclusions. Significantly, similarly to what we observed in tissue culture cells, p300 also co-localized with ubiquitin and with HDAC6 in the LBs of affected patients (**Figure 2D** and **Figure 2E**).

Thus, p300 is a component of aggresomes and of pathological aggregates typical of Parkinson Disease.

### The C-terminus of p300 is Necessary for p300 Localization into Aggresomes

**Figure 3:**
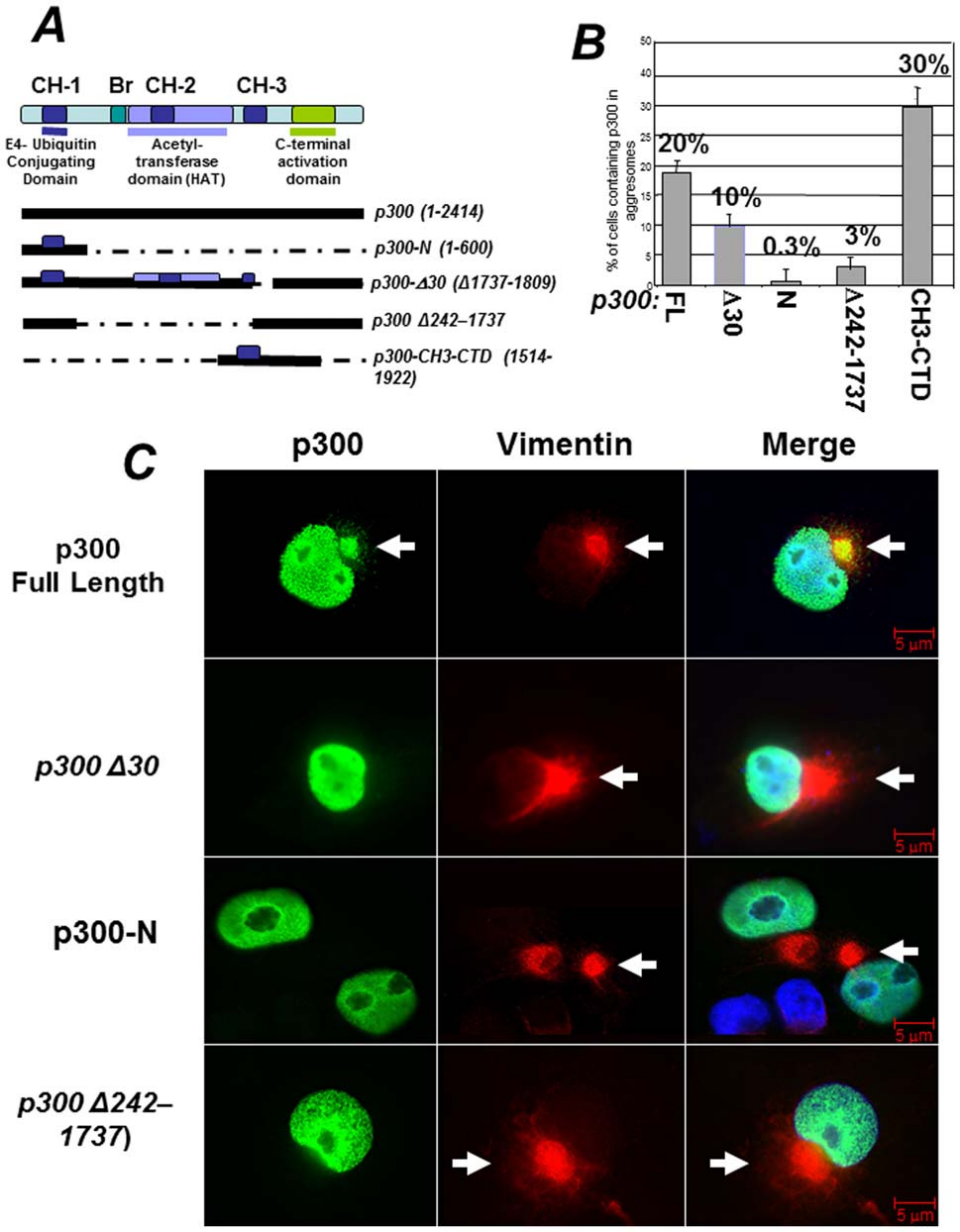
Identification of the structural domain of p300 responsible for localization into aggresomes. **A**. Schematic representation of the various p300 domains (see also text for further description). The three Cystein-Histidine-Rich domains, (CH1, CH2 and CH3), are delineated by colored boxes. The bromodomain (Br) and histone acetylatransferase domains (HAT) are labeled by colored horizontal lines. Under the diagram, a scheme of the p300 expression vectors and relative amino acid residues affected by deletions/mutations used for detection of p300 in aggresomes, is shown. **B.** Quantification of the immuno-fluorescence experiments, showing the percent of cells containing p300 in aggresomes relatively to the total number of cells expressing each of these deletion products. Cos7 cells were transfected with the vectors expressing p300 full-length, p300Δ30 or p300-N, p300-Δ242–1737 or p300-CH3-CTD and the percentage of cells containing p300 in aggresomes was calculated from two separate experiments. Representative immuno-fluorescence data from these experiments are shown in panel **C**. **C**. Cells were treated with MG132, and subsequently stained for vimentin (red), or for p300 and then counterstained with DAPI (only shown in the merge panels). Arrows indicate the position of aggresomes.

p300 is a large protein with multiple functional domains (depicted in **Figure 3A**) [16]. There are three histidine rich motifs in p300, named CH1, CH2 and CH3, each containing zinc-fingers domains (ZNFs) with different structural and functional characteristics. The region comprising the CH1 is well characterized and possess an E4-ubiquitin elongating activity that aids in the degradation of p53 [19], [20], [23]. The CH3 encompasses two structurally distinct zinc fingers (ZNF) the function of which has not been entirely elucidated. The first ZNF, ZNF-ZZ spans between amino acids 1684–1703, while the second, ZNF-TAZ2, is comprised between amino acid 1723–1836. The histone acetyltransferase domain (HAT) maps to a large central fragment of the protein, with the core segment comprised between amino acids 1284–1669. To determine what region of p300 is involved in the association with aggresomes we examined the localization pattern of several deletion mutants differing for the presence or absence of these structural motifs (quantified in **Figure 3B** and shown as representative immuno-fluorescence data in **Figure 3C**). As shown previously, full-length p300 was detected in aggresomes. By contrast a p300 N-terminal fragment containing the first CH1 and encompassing the previously identified E4-ubiquitin domain (p300-N) was never found in the cytoplasmic compartment nor was it localized in aggresomes. Therefore, the association of p300 with aggresomes and ubiquitin that we observed previously is independent of this domain. Consistent with this interpretation, a p300 deletion mutant possessing the CH1 but lacking the acetyltransferase domain and the first ZN-FF zinc finger within the CH3 (p300 Δ242–1737) displayed impaired localization into aggresomes, and a nearly full-length p300 protein with a small deletion of the second zinc finger in the CH3, TAZ2 (p300-Δ30, or Δ1737–1809), was found in these organelles to a significant lesser extent compared to native p300. Finally, a p300 protein encompassing the entire CH3 and most of the p300 C-terminus (from amino acids 1514 to 1922, referred to as CH3-CTD), efficiently localized into aggresomes (**Figure 3B**), thus suggesting that this domain might be responsible for routing p300 into these organelles.

### The C-terminus of p300 is a Disordered Domain with Similarities to Prion-like Domains

**Figure 4:**
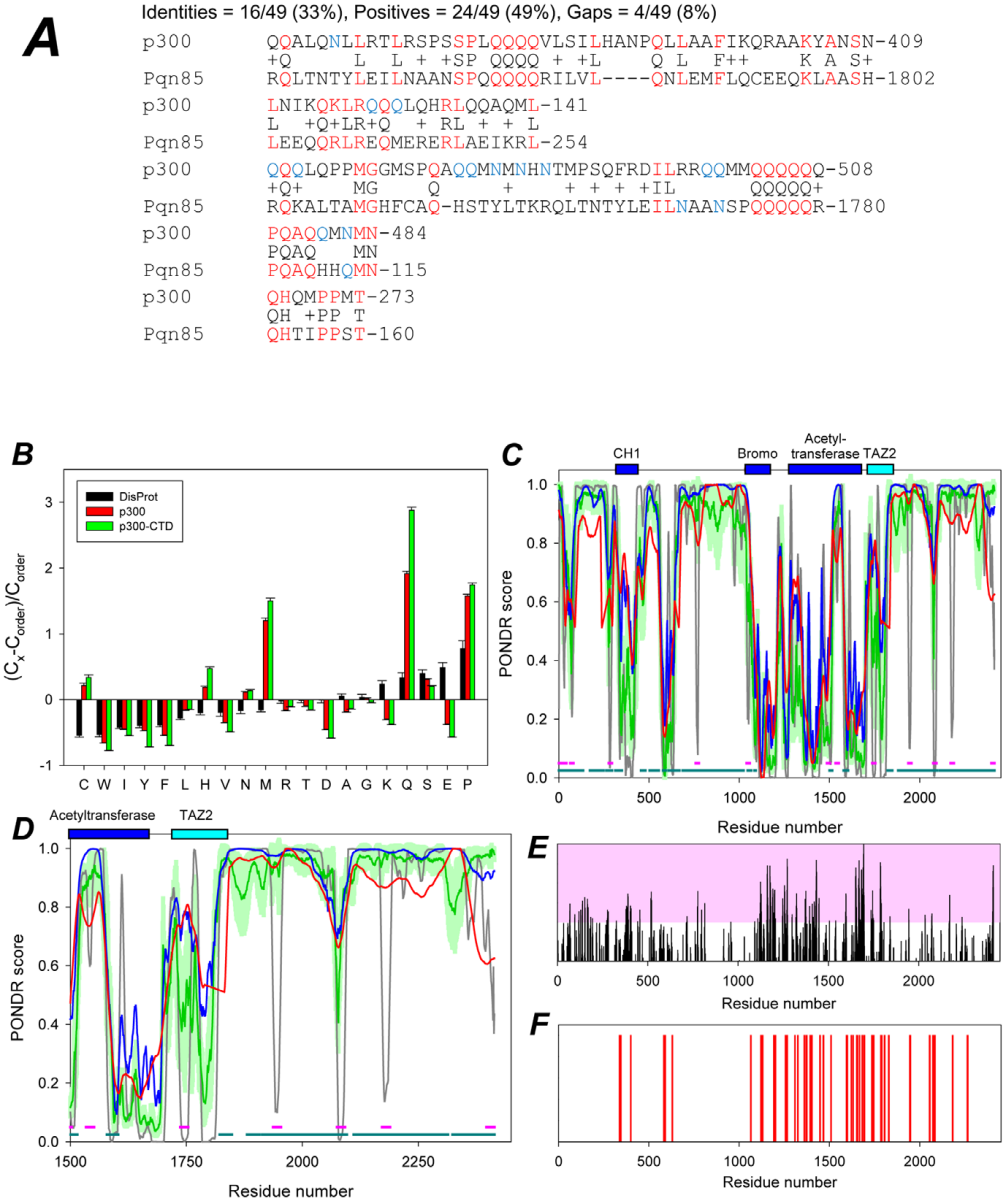
*In silico* analysis of the region of p300 responsible for the localization into aggresomes. A. Blast results of the region of p300 encompassing amino acid residues 1514 to 2414 showing representative similarities with the prion-like protein pqn-85 of *C.Elegans*. The results of this search are displayed in more detail in **table S1**. Amino acid residues labeled in red designate identity, while the blue color outlines the Q and N residues in the p300 or pqn-85 proteins. p300 has a 22% total content of Q/N in this region **B.** Compositional analysis of full length p300 (red) or of the p300-CTD (green) in comparison with composition of typical ordered proteins. Compositional profile of typical intrinsically disordered proteins from the DisProt database is shown for comparison (black bars). Positive bars correspond to residues found more abundantly in p300, while negative bars show residues, in which p300 is depleted. This analysis shows that compared to ordered proteins, p300 is enriched in the disorder-promoting residue C, H, M, Q, S, N, and P and is depleted in major order-promoting residues W, F, I, Y, V, L, A, R, D, E and K. Furthermore there is a low content of charged residues, with enrichment in polar but non-charged residues. **C–D**. Evaluation of intrinsic disorder in the full-length p300 (**C**) and its C-terminal domain (CTD), residues 1514–2414 (**D**). Four disorder prediction tools of the PONDR family were used (28–30): PONDR® VSL2B, which is statistically better for proteins containing both structure and disorder; PONDR® VL3 which is better for proteins that are experimentally known to be 100% disordered or possess long disordered regions; PONDR® VLXT which is useful for predicting MoRFs, short disordered regions that become structured when they interact with their binding partners; and PONDR-FIT a meta-predictor which is statistically not different from PONDR® VL3 for fully disordered and fully structured proteins, and slightly better (1 std) than PONDR® VSL2 when both structured and disordered regions are present. The dark gray lines are disorder predictions by PONDR®VLXT; the red lines represent results of disorder prediction by PONDR®VL3; the blue lines show disorder predictions by PONDR®VSL2; whereas green lines correspond to the results produced by PONDR-FIT; light green shadows represent standard errors of disorder prediction by PONDR-FIT. Locations of structured domains (CH1 domain, residues 323–423 (PDB IDs: 1L3E and 1P4Q); bromo-domain, residues 1040–1161 (PDB ID: 3I3J); acetyltransferase domain (HAT), residues 1284–1669 (PDB ID: 3BIY); and TAZ2 domain, residues 1723–1836 (PDB ID: 3IO2) are shown by dark blue and cyan bars. Figure 4C and 4D show that these proteins are highly disordered, since their curves are mostly located above 0.5, albeit they also contain ordered regions. Figure 4C also shows that p300 contains a large number of potential disorder-based binding sites, α-MoRFs and ANCHOR-indicated binding sites (AiBSs) (29–30). Locations of α-MoRFs and AiBSs are shown as pink and dark cyan bars at the bottom of plots.

The above findings prompted us to ask whether the p300 C-terminus possesses distinguishable structural motifs common to other proteins involved in protein aggregation. A BLAST search of the p300 amino acid sequences encompassing amino acid residues 1688 to 2414 revealed the existence of a significant homology with various prion-like proteins expressed in various species, including *C. elegans,* which aligned with p300 with a highly significant Blast e-value (>0.001) (**Figure 4A** **and Table S1**). In an interesting analogy, some of these *C. elegans* genes, the *Abu* family, were isolated in metazoans carrying mutations that hamper the folding capacity of the endoplasmic reticulum (ER) [23], a situation that overwhelms cells with misfolded proteins in a manner similar to that achieved with proteasome inhibitors. Based on these similarities, we call this domain, **p**300 region **S**imilar to **P**rion-like **D**omains, or PSPD.

Several lines of evidence indicate that prion proteins contain intrinsically disordered regions. For example, the yeast prion proteins Ure2p and Sup35p contain a prion domain in their N-terminus which is sufficient for prion formation and is intrinsically disordered [24]. Similarly, toxic signaling due to the cellular PrP^C^ protein requires the intrinsically disordered N-terminal domain [25], [26]. Thus, we next studied the intrinsic disorder propensity of both the full length p300 protein and of the PSPD (1514–2414), by analyzing their compositional profiles. This analysis is based on the important observation that amino acid compositions of ordered and intrinsically disordered proteins are very different, with disordered proteins being systematically enriched in so-called disorder-promoting residues (A, R, G, Q, S, E, K, and P) and depleted in order-promoting residues (W, Y, F, I, L, V, C, and N) [27], [28], [29]. Composition profiling is based on the evaluation of the (C_s1_–C_s2_)/C_s2_ values, where C_s1_ is a content of a given residue in a protein of interest (p300 or its C-terminal domain), and C_s2_ is the corresponding value for the sample set of ordered proteins from the public domain binding database, PDB [29]. Relatively to typically ordered proteins, p300 and the PSDP are depleted in major order-promoting amino acids (W, F, I, Y, V, and L) and are enriched in some disorder-promoting residues, particularly Q, S, and P (**Figure 4B**). Interestingly, both p300 and the PSPD have low content of charged residues, being instead enriched in some polar but non-charged residues. **Figure 4C** and **Figure 4D** represent evaluation of disorder propensity in these proteins studied by employed four disorder prediction tools of the PONDR family. This analysis showed that full length p300 and especially its C-terminal region are highly disordered. At the next stage, two different algorithms were used to find disordered regions of p300 that can bind to specific partners, and potentially undergo the disorder-to-order transitions as a result of this binding. **Figure 4C** shows that p300 contains a large number of potential disorder-based binding sites, α-MoRFs [30], [31], and ANCHOR-indicated binding sites (AiBSs) [32], which often completely or partially overlap with each other. The presence of these sites is an indication that the major function of the disordered p300 domains might consist in providing an interaction platform with various binding partners, a view supported by results presented later.

### The PSPD Contains Significant Amount of Aggregation-prone Regions

Several algorithms for predition of the aggregation-prone regions within a protein of interest are available. Here, we utilized two of such algorithms, FoldAmyloid (http://antares.protres.ru/fold-amyloid/), and Zyggregator (http://www-vendruscolo.ch.cam.ac.uk/zyggregator.php). These predictors use the knowledge of the sequence of amino acids for the simultaneous estimation of both the propensity for folding and aggregation and the way in which these two types of propensity compete [33], [34]. Application of these algorithms in the early large-schale studies revelated that the regions of a protein with a high intrinsic aggregation propensity can be identified in a robust manner from sequence only and also clearly showed that the structural context of such regions in the monomeric protein prior the aggregation is crucial for determining their actual role in the aggregation process [33], [34]. Of special interest is the early observation that the protein regions with high expected probability of the formation of backbone–backbone hydrogen bonds as well as regions with high expected packing density are mostly responsible for the formation of amyloid fibrils [33], especially if these regions are located within the intrinscially disordered proteins/domains. The results of the evaluation of the aggregation propensity of different region of p300 by Zyggregator and FoldAmyloid are shown in **Figures 4E and 4F**, respectively. This analysis revealed that the aggregation-prone regions are predominantly located within the central and C-terminal domains of p300. Furthermore, 18 of 38 amyloidogenic regions predicted by FoldAmyloid are located within the PSPD, whereas N-terminal domain (residues 1–900) contains just 6 such regions. Longest aggregation-prone regions (residues 1733–1747 and 1783–1792) are also located in PSPD. Finally, according to FoldAmyloid, of 900 residues in PSPD, 126 were included into the aggregation-prone regions. In combination with the fact that PSPD was predicted as mostly disordered domain, these observations suggest that the p300 region similar to prion domains play a crucial role in the aggregation behavior of this protein.

### The PSPD is Encoded as an Alternatively Spliced Variant and Promotes the Association with Aggresomes and with Ubiquitinated Proteins

**Figure 5:**
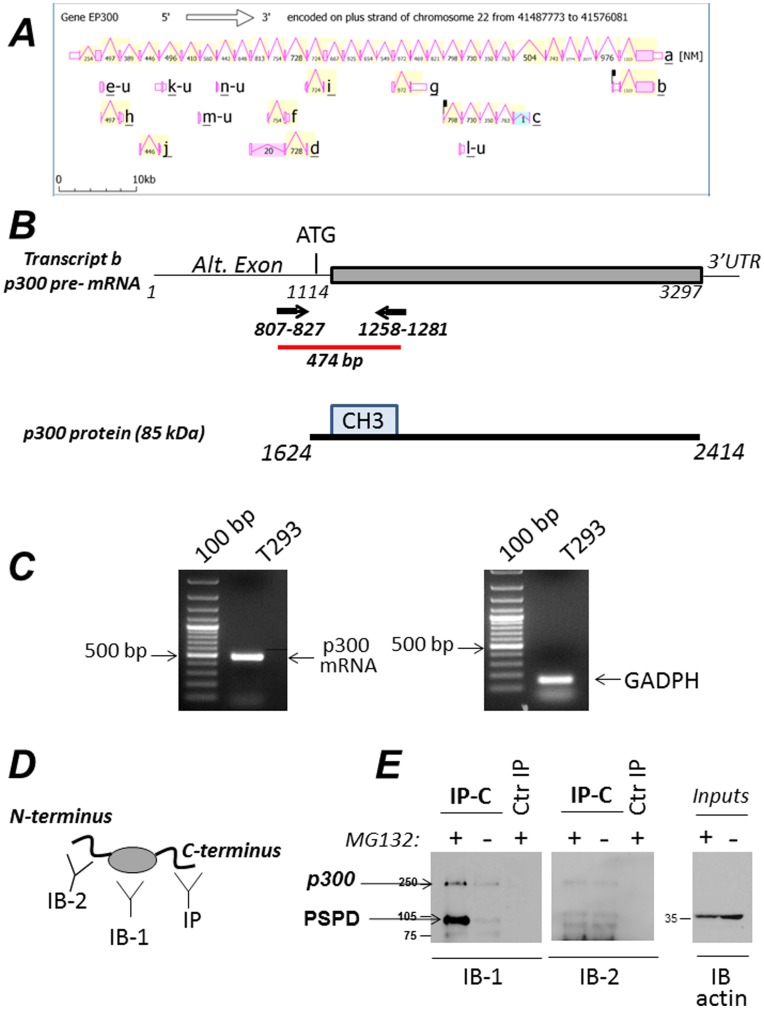
An alternatively spliced product of p300 encompasses the PSPD region and is enriched in MG132 treated cells. **A.** Schematic representation of the alternative exons and spliced variants of p300. These data were extracted from the Aceview database (31,32). **B**. Structure of the pre-messenger mRNA (variant b, http://www.ncbi.nlm.nih.gov/IEB/Research/Acembly/av.cgi?db=human&term=EP300&submit=Go). This complete CDS mRNA is 3297 base pair long. It is predicted to encode a protein of 85 kDa which is identical in sequence to the full length p300, encompassing amino acid residues 1624 to 2414. To gain evidence for the presence of this mRNA, we employed a combination of primers localized in the alternative exon (depicted in panel B). As shown in panel **C** (left panel), an product of expected size is detected in Hek293 cells after reverse transcription using oligo-dT primer 5′. This PCR product was sequenced and matched the sequences of the p300 mRNA. The right panel in C, shows a control amplification reaction with GADPH primers. The position of the amplified products relatively to the 100 bp ladder is indicated. **D–E.** Detection of a p300 polypeptide lacking the N-terminal region and encompassing the p300 C-terminus. Panel D illustrates the strategy employed for these experiments. Cellular extracts derived from Hek293 untreated or MG132 treated were equalized for protein concentration, and first immuno-precipitated with the anti-p300 antibody directed against the C-terminal region (C20, SC). The product of these immuno-precipitation reactions were then probed in immuno-blot with an antibody directed against either amino acid residues 1572–2371 (RW109; indicated as IB-1), or with antibody raised against the N-terminal region (N15; indicated as IB-2). As shown in panel E, a p300 product of compatible size is detected and enriched in MG132 treated cells in the IB-1 immuno-blot (left panel), but not in the IB-2 IB (mid panel). The right panel shows the inputs levels of cell extracts employed for the immuno-precipitations with an equal actin signal. Arrows indicate the presence of p300 full length or of the PSPD.

During the course of our analysis of p300 levels in cells, we detected the presence of various p300 protein species with various electrophoretic mobility (i.e., see **Figure 5E**). The difference in the molecular weight of these p300 isoforms suggested that they might represent alternatively spliced products. To explore this possibility, we first interrogated the Aceview database at NIH, which provides a curated and comprehensive collection of all recorded mRNA sequences [35], [36]. Analysis of the p300 gene organization suggests the existence of at least 9 mRNA variants (**Figure 5A**). Significantly, transcript *b* encodes a truncated version of p300 lacking the N-terminus the CH1, CH2 and the HAT domain, and encompassing almost entirely the PSPD region from amino acids 1624 to 2414 (**Figure 5B**). The existence of this complete and capped mRNA is supported by various clones and can be seen at http://www.ncbi.nlm.nih.gov/IEB/Research/Acembly/av.cgi?db=human&term=EP300&submit=Go). The alternative exon of transcript *b* is predicted to encode a protein of 85 kDa with no sequence dissimilarity relatively to the main p300 transcript. To gain further evidence for the existence of this mRNA, we designed a combination of primers localized in the alternative intron-exon junction (schematically shown in **Figure 5B**). A mRNA product of expected size is detected in Hek293 cells after reverse transcription using an oligo-dT primer 5′ (**Figure 5C**). To then determine whether this mRNA generates a protein product, cellular extracts derived from Hek293 untreated or MG132 treated cells were first immuno-precipitated with the anti-p300 antibody directed against the last C-terminal portion (Ab-C20) (see strategy illustrated in **Figure 5D**). The products of these immuno-precipitation reactions were then probed in immuno-blot with an antibody raised against amino acid residues 2107–2283 (Ab-RW109; indicated as IB-1 in **Figure 5D**) or with an antibody raised against the N-terminal region (Ab-N15; indicated as IB-2). As shown in **Figure5E**, a p300 product of lower molecular weight was detected and was strongly enriched in MG132-treated cells in the IB-1 immuno-blot (left panel, indicated by arrow), but not in the IB-2 IB (mid panel). Because the RW109 antibody recognizes the p300 C-terminus but does not interact with the N-terminus or with the HAT domain, these results demonstrate the existence of a p300 polypetide corresponding to the PSPD.

**Figure 6:**
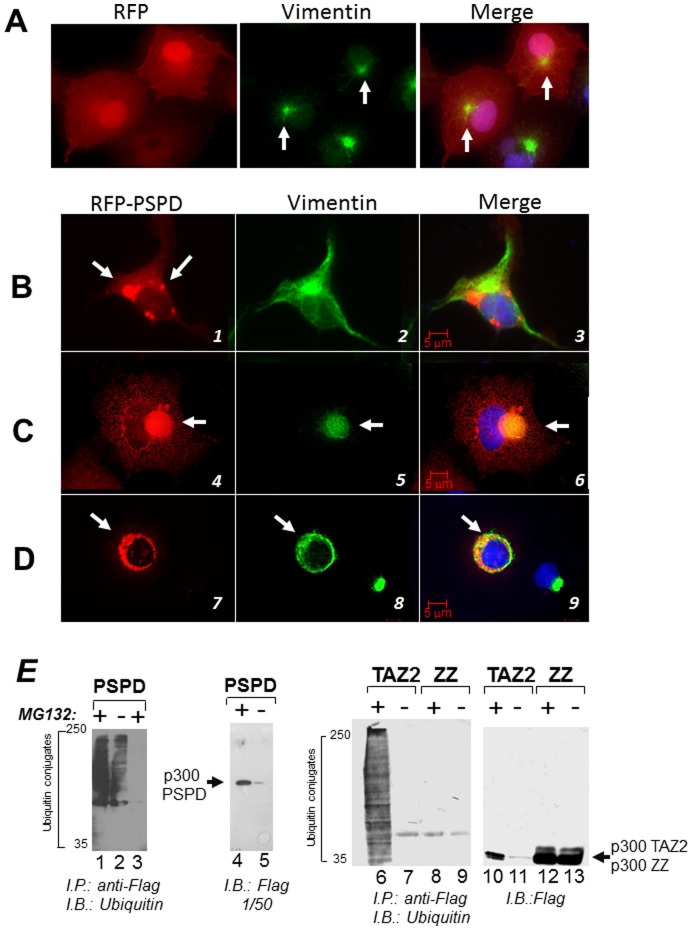
The p300 PSPD is sufficient for aggresome localization. **A–D.** Cell transfected with the vector expressing RFP alone and treated with MG132 (panel A), or transfected with the RFP-PSPD (panels B–D) were mock treated (B), or treated with MG132 (**C–D**) and subsequently stained for vimentin (green). Panels **C** and **D** contain two representative fields of cells transfected with RFP-PSPD. Arrows indicate the position of aggregates formed in the absence (panel B) or in the presence of MG132 (C and D). **E.** Interaction of p300 with ubiquitinated protein species. The epitope-flagged vector expressing the PSPD (lanes 1–5), or the TAZ2 (lanes 6,7 and 10,11) or the ZZ (lanes 8,9 and 12,13) were transfected in Cos7 cells. Cells were left untreated (−) or treated with MG132 (+), cell extracts were immuno-precipitated with the anti-Flag antibody (lanes 1,2; and 6-to-9), or with a control isotype matched antibody (lane 3) and the products of these immuno-precipitation reactions were probed with ubiquitin antibodies as indicated at the bottom of each panel. Alternatively, the anti-Flag immuno-blot on total cell extracts shows the total amount of p300 proteins present in these reactions, which were derived from approximately 1/50 of total extracts.

To then assess the function of this alternatively spliced product, we studied the activity of a large p300 fragment encoding amino acids 1514–1922, which encompasses almost entirely the PSPD. First, we asked whether the PSPD, *per se*, is able to promote aggregation and localization into aggresome of a foreign protein (Red Fluorescent Protein, RFP). As shown in **Figure 6**, in MG132 treated cells RFP alone was seen predominantly in the nucleus and diffusely in the cytoplasm (**Figure 6A**). Expression of PSPD re-localized RFP into disperse cytoplasmic inclusions in the absence of MG132 (**Figure 6B**, compare panels 1 and 2 with panel 3). Furthermore, in MG132 treated cells RFP-PSPD formed larger aggregates that exhibited two different patterns of localization: it either formed distinct and large aggresomes with a well-defined collapsed vimentin ring (**Figure 6C** **,** panels 4 to 6); or it co-localized, together with vimentin, in large inclusions that occupied the cytoplasm and surrounded the nucleus (**Figure 6D**, **7** to **9**). Time-lapse video-microscopy performed in cells transfected with RFP-PSPD demonstrated that this ring structure represents an initial stage of aggresome formation, which ultimately leads to the defined juxta-nuclear bodies (**movie S1**). These observations demonstrate that the p300 PSPD accompanies all stages of aggresome maturation, and suggest that p300 might be a structural component of these organelles.

Misfolded proteins that accumulate in aggresomes are poly-ubiquitinated. In keeping with the above results, and given the previously shown association of p300 with ubiquitinated protein species, we then asked whether the PSPD is responsible for driving the interaction with ubiquitinated polypeptides. To test this, the vector expressing the p300-PSPD tagged with a Flag-epitope was expressed in Cos7 cells in the presence or absence of MG132, and subjected to the immuno-precipitation with the anti-Flag antibody followed by immuno-blot with anti-ubiquitin antibodies. Moreover, we used two additional constructs containing either the TAZ2- or the ZZ- zinc fingers of p300. In the immuno-precipitation reaction containing the PSPD and TAZ2 domain of p300, but not the ZZ finger, multiple ubiquitinated polypeptides were clearly detected (**Figure 6E**, compare lanes 1, 2 and 6 with lanes 8 and 9). These species did not react with the anti-flag antibody (**Figure 6E** **,** lanes 4 and 5; and lanes 10 to 12), thus demonstrating that they do not correspond to ubiquitinated forms of p300 itself, but rather reflect the p300 association with ubiquitinated protein species. Therefore, within the PSPD, the TAZ2 domain promotes the association with ubiquitinated proteins.

### The p300 PSPD Promotes Aggregation of TAU, of p53 and of p21/WAF

The presence of intrinsically disordered domains within a protein enhances interaction interfaces, functional complexity, as well as the propensity to associates promiscuously with other disordered proteins [37], [38]. Therefore, we first examined whether p300 interacts with TAU, whose aberrant conformations and aggregation hallmark Alzheimer’s disease and other neuropathologies referred to as tauopathies [39]. Co-localization of TAU and endogenous p300 was detected in TAU typical cytoplasmic filaments in approximately 10% of the cells expressing TAU (**Figure S3A and S3D, panel** ***3***). Furthermore, transfection of the p300 PSPD led to a change in the pattern of TAU distribution in cells, with a significant increase in the percentage of cells displaying TAU cytoplasmic aggregates (**Figure S3B** and **S3D, panel 6,** and quantified in **panel S3C**). Indeed, in cells transfected with control vector 30% of cells formed aggregates, but almost all (approximately 80%) of cells co-transfected with the PSPD displayed an aggregated pattern.

**Figure 7:**
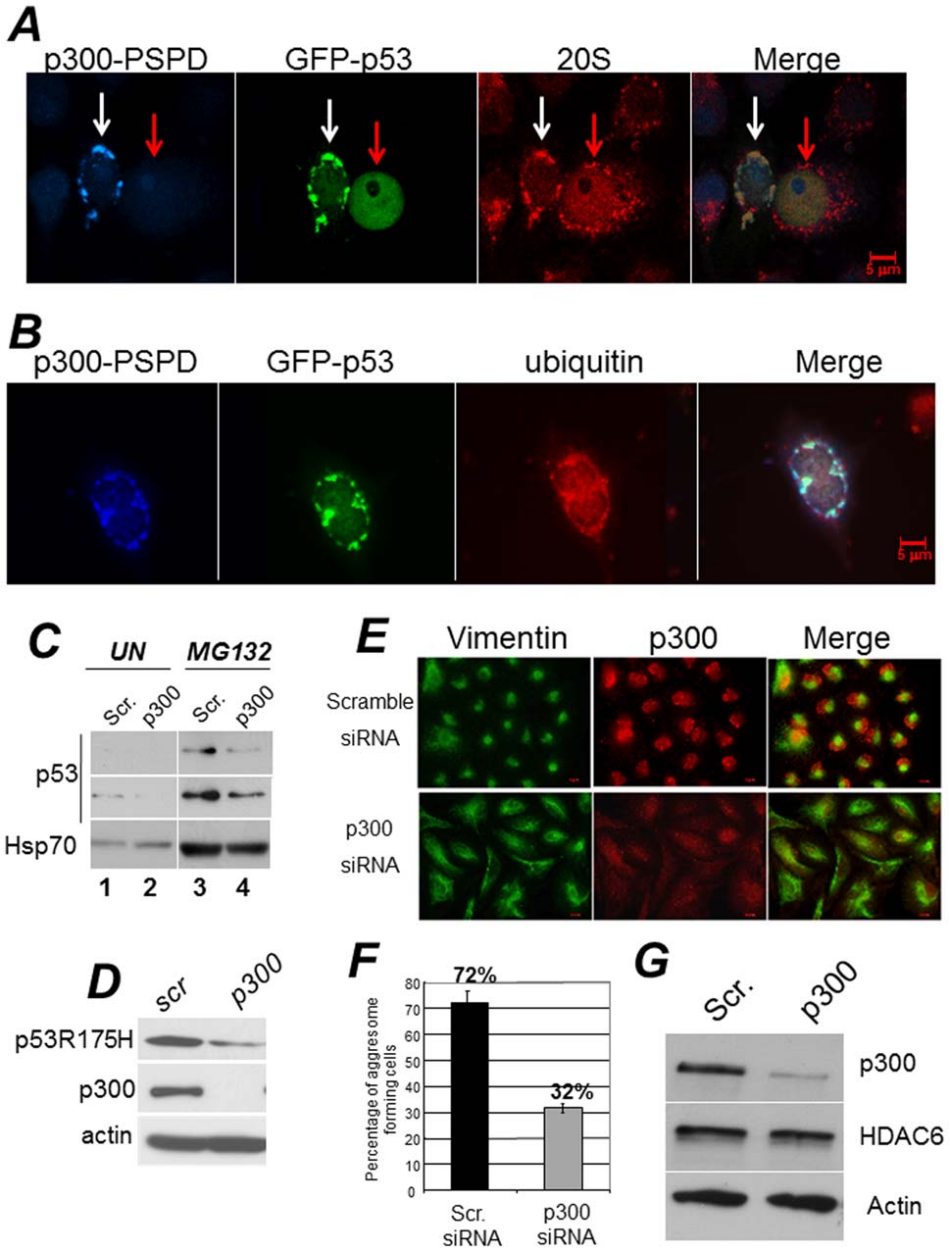
A-B. The p300-PSPD brings p53 in cytoplasmic aggregates. Hek293 cells were co-transfected with a vector expressing epitope tagged GAL4-PSPD and p53. In panel **A** cells were stained with an antibody recognizing p53 (polyclonal goat, blue), with anti-GAL4 antibody (monoclonal mouse, green), and with the anti-20S proteasome polyclonal antibody (red). The white or red arrows indicate cell expressing or not p300-PSDP. Note that p53 forms inclusions only when p300-PSDP is expressed. In panel **B**, a vector expressing GFP-p53 was employed to demonstrate co-localization with the 20S subunit of the proteasome (red). Merged images are shown in the last right panel. p53 did not form inclusions with control vector alone (not shown). **C.** A549 cells e were transfected with scramble, control siRNA (lanes 1 and 3), or with the p300 specific siRNA (lanes 2 and 4). Seventytwo hours after transfection cells were treated with vheicle (lanes 1, 2) or with 20 µM MG132 (lanes 3,4) for 16 hours, and cell extracts were prepared. Total levels of p53 and Hsp70 are shown. **D.** TOV cells expressing the tumor-derived p53 mutant, p53R175H, were transfected with scramle or p300 shRNA as described in C, and cell extracts derived from these transfection were probed for p53, p300 and actin. **E–F.** **The knock-down of p300 impairs aggresome formation. E.** A549 cells were transfected with scramble siRNA or with the p300 specific siRNA. Cells were grown on glass cover slips, treated with MG132 for 16 hours, and then probed with the anti-p300 specific polyclonal antibody (red), or with the monoclonal antibody directed against vimentin (green). Representative fields are shown in D. **F.** Quantification of the experiments shown in E. Aggresomes were counted in cells transfected with scramble- or p300-specific siRNA and percentages are shown at the top of the bars. Black and gray bars indicates the percentage of cells displaying aggresomes in the presence of control or p300 siRNA. The presence of aggresomes was assessed based on the presence of the vimentin ring. Results are representative of two independent experiments, each performed in duplicate. **G.** Assessment of HDAC6 expression levels in A549 cells transfected with control- and p300-siRNA.

The tumor suppressor p53 has an intrinsically disordered conformation and although is a predominantly nuclear protein, its abnormal localization in the cytoplasm in a misfolded configuration often in the form of inclusions, occurs in various tumors [40], [41], [42]. The tendency to aggregate is increased by tumor derived mutations of the p53 gene within the DNA binding domain and depends, in part, upon their defective clearance [43]. These considerations led us to examine whether p300 affects p53 aggregation. First, we found that endogenous wild-type p53 co-localized with p300 and with the 20S subunit of the proteasome in cytoplasmic aggregates of Hek293 (**Figure S3E and S3F**), where p53 accumulates due to the presence of adenoviral proteins E1a and E1B [44]. Second, expression of the p300-PSPD was able to direct p53 localization into aggregates that contain ubiquitin and the 20S subunit of the proteasome (**Figure 7A and 7B**). These results suggested that by inducing aggregation, p300 might sequester p53 from proteasomal degradation. To further explore this possibility and to rule out cell type-specific effects, we transfected H1299 cells expressing wild-type p53 with various p300 constructs (**Figure 3**). In H1299 cells p53 is predominantly nuclear and transfection of the p300-Δ30 or of the p300-N did not modify this pattern of localization (**Figure S4A and S4B**). By contrast, expression of the p300-PSPD relocalized p53 into disperse cytoplasmic aggregates in untreated cells and in large cytoplasmic inclusions in cells treated with MG132 (**Figure S4C and S4D**). Significantly, the knock-down of p300 in A549 cells lowered p53 levels and prevented stabilization induced by MG132 (**Figure 7C**). Since p300 also interacts with other tumor suppressors [43], [45], including p21/Waf, we next asked whether the PSPD can induce aggregation of p21 as well. As shown in **Figure S4E**, while p21 dysplays a predominantly nuclear pattern of localization, in cells expressing the p300-PSPD p21 was relocalized into discrete cytoplasmic aggregates.

It has been recently shown that misfolded, tumor derived mutant forms of p53 can aggregate in various tumor cell lines correlating with enhanced oncogenic capacity [40], [41], [43]. Thus, we expanded these experiments to ask whether p300 affects p53 mutant aggregation. We found that expression of the p300-PSPD induced aggregation of two different tumor derived mutants, p53H175R and p53G245R (**Figure S5**), while down-regulation of p300 in TOV cells, that express endogenous p53H175R, destabilized mutant p53 (**Figure 7D**).

These results suggest that *via* the PSPD, p300 can bring TAU, p21 and p53 into cytoplasmic inclusions leading to aggregation and at least in the case of p53, to stabilization.

### p300 is Necessary for Segregation of Ubiquitinated Proteins into Aggresomes and Promotes Survival during Misfolded Protein Stress

**Figure 8:**
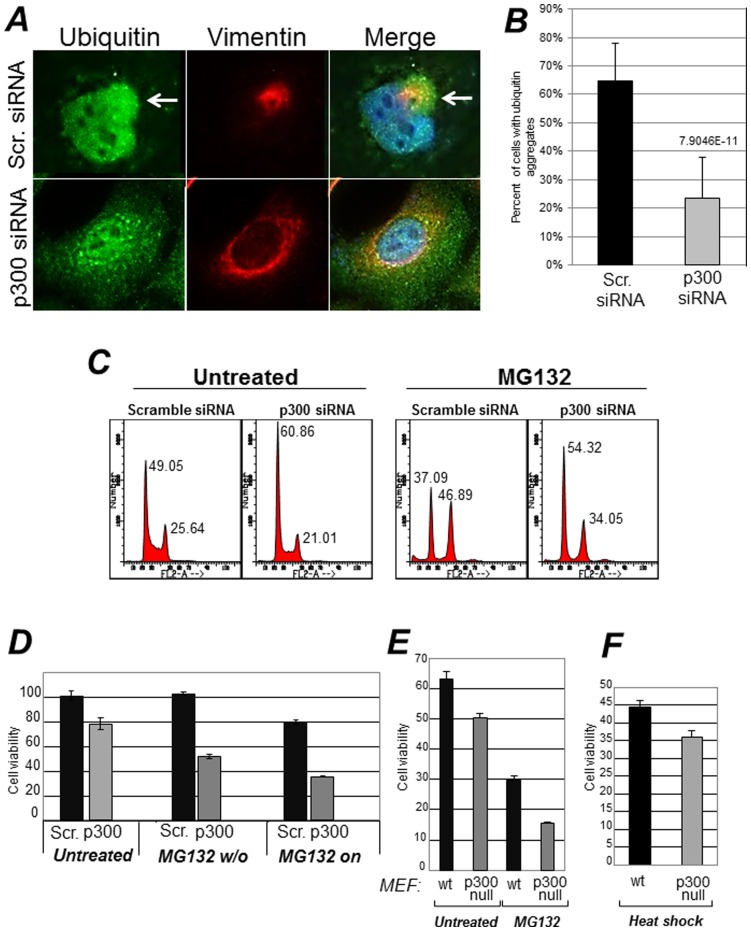
Effects of p300 on the misfolded protein response. A. A549 cells were transfected with scramble siRNA or with the p300 specific siRNA as described previously, and stained with the anti-ubiquitin (green) or anti-vimentin (red) antibody. The percentage of cells containing ubiquitin aggregates is quantified in panel **B**. **C**. A549 cells were transfected with the scramble- or p300-specific siRNAs were mock treated or treated with MG132 for 16 hours, then harvested for flow cytometry. Panel C shows the Propidium Iodide profiles, and percentages of cells in the G1 or G2 phases of the cell cycle are indicated at the top of each relative peak. **D.** A549 cells transfected with scramble (black bars) or with the p300 siRNAs (gray bars), were treated with MG132. In one set of samples MG132 was washed out after 12–14 hours of treatment (indicated as MG132 w/o) and cells were allowed to recover for about four days, at which time they were counted. Alternatively, cell growth was monitored for the same period of time in the presence of MG132 (indicated as MG132 on). Error bars represent standard deviations. **E–F.** Control (wt) or p300 null mouse embryo fibroblasts (MEF) were mock treated or treated with MG132 for 16–24 hours (panel E), or alternatively, subjected to Heat Shock (HS, panel F) by incubating the cells at 40°C for two hours. Cells were allowed to recover from MG132 treatment or HS for 24–48 hours and were counted. Cell viability was assessed with trypan blue exclusion.

We showed previously that p300 localizes into aggresomes, and that expression of the PSPD leads to detection of intermediate precursor forms of these organelles, suggesting that p300 participates in some aspect of aggresome formation and/or clearance. To test this hypothesis, we first studied the extent of aggresome formation in cells were p300 levels were down-modulated. While the majority of A649 cells receiving a control siRNA formed aggresomes, these structures were detected in only a very small percentage of cells harboring the p300 siRNA (72% *versus* 32% respectively) (**Figure 7E** and quantified in **Figure 7F** **)**. This was not due to down-regulation of HDAC6, which is essential for the formation of these structures, because HDAC6 levels were unchanged (**Figure 7G**). Furthermore, in cells where p300 was down-regulated, there was an altered localization pattern of ubiquitinated protein species, that remained dispersed in the cytoplasm and failed to form typical aggregates as in control cells (**Figure 8A and B**).

Aggresomes have cytoprotective effects during misfolded protein stress [8]. This protective role has been attributed to the segregation of misfolded and ubiquitinated proteins that prevents them from exerting potential toxicity in the cytoplasm or in other compartments of the cell. Therefore, we next investigated whether p300 modulates cellular susceptibility to this response. A549 cells were transfected with either the control or with the p300 siRNA and subsequently were treated with MG132. We observed that while MG132 treatment induced an arrest in the G2 phase of the cell cycle in control transfected cells, cells where p300 was inhibited with the specific siRNA were only modestly arrested in G2 (**Figure 8C**). More significantly, in cells expressing p300, a short-term treatment with MG132 for 12 hours followed by a wash-out did not compromise cell viability, while cells effectively depleted of p300 completely failed to recover after removal of the drug (**Figure 8D**, indicated as MG132 w/o). Similar experiments were next performed in p300 null mouse embryo-fibroblasts (p300−/−MEF) [46], and identical results were obtained (**Figure 8E**). To ensure that lack of viability is not due to a general and non-specific hyper-sensitivity of cells lacking p300 to all forms of stress, we assayed the sensitivity of p300−/−MEF to heat shock (HS). This type of stress also induces misfolding and denaturation of intracellular proteins, however, cells rely for survival on the folding capacity of the ER and on *de novo* protein synthesis [47]. Unlike in the case of proteasomal inhibition, p300−/− and wild-type MEF displayed similar rates of survival during HS (**Figure 8F**). Thus, these results indicate that p300 acts as a survival factor during the misfolded protein response due to proteasomal stress.

## Discussion

In this study we have shown that p300 possesses an intrinsically disordered domain, which is capable to direct p300 localization into cytoplasmic aggregates. Intriguingly, this domain shares similarities with prion-like proteins including the *Abu* family of genes of *C. Elegans* [23]. *Abu* genes were found up-regulated in worms with hampered ER activity that leads to an overload of incorrectly folded proteins. In a compelling analogy, in *C. Elegans Abu* genes are required for the ability to cope with such surplus of misfolded proteins, and in mammalian cells p300 functions as a pro-survival factor when aberrant proteins accumulate as a result of proteasome block. Thus, like *Abu* genes, p300 helps cells deal with stress arising from accumulation of misfolded proteins.

### Proposed Model of Action of the p300 PSPD

Although the p300 PSPD is not a canonical prion domain, it is worth discussing several of its characteristics that insinuate functional analogies with prion-like regions. Prion domains have a particularly high content of glutamine and asparagine residues and induce self- and hetero-aggregation [48]. The PSPD possesses an unusually high Q/N content (22%), it forms aggregates, -suggestive of self-aggregation-, and is also capable of inducing aggregation of TAU and of p53. Furthermore, the *in silico* analysis revealed that this p300 domain is enriched in the aggregation-prone regions (**Figure 4E and 4D**). Intriguingly, recent evidence demonstrates that some co-repressors and components of the transcriptional machinery, such as SWI/SNIFF and Cyc8 contain aggregation prone domains and can replicate as prions *in yeast* [49], [50]. Moreover, there are other examples of proteins characterized by glutamine rich regions that confer high propensity to aggregation, and thus mimic aggregation prone elements of prions even though they are devoid of infectious properties. The disease-associated huntingtin protein possesses an extended glutamine tract that drives its aggregation in Hungtington Disease. Curiously CBP, but not p300, displays a poly-glutamine tail in its C-terminal region that allows it to interacts with huntingtin in typical inclusion bodies [51]. However, unlike aggregates formed by p300 that reside in the cytoplasm, inclusion bodies formed by CBP are nuclear. Thus, both p300 and CBP have aggregation-prone elements, however they form aggregates in different sub-cellular compartments.

**Figure 9:**
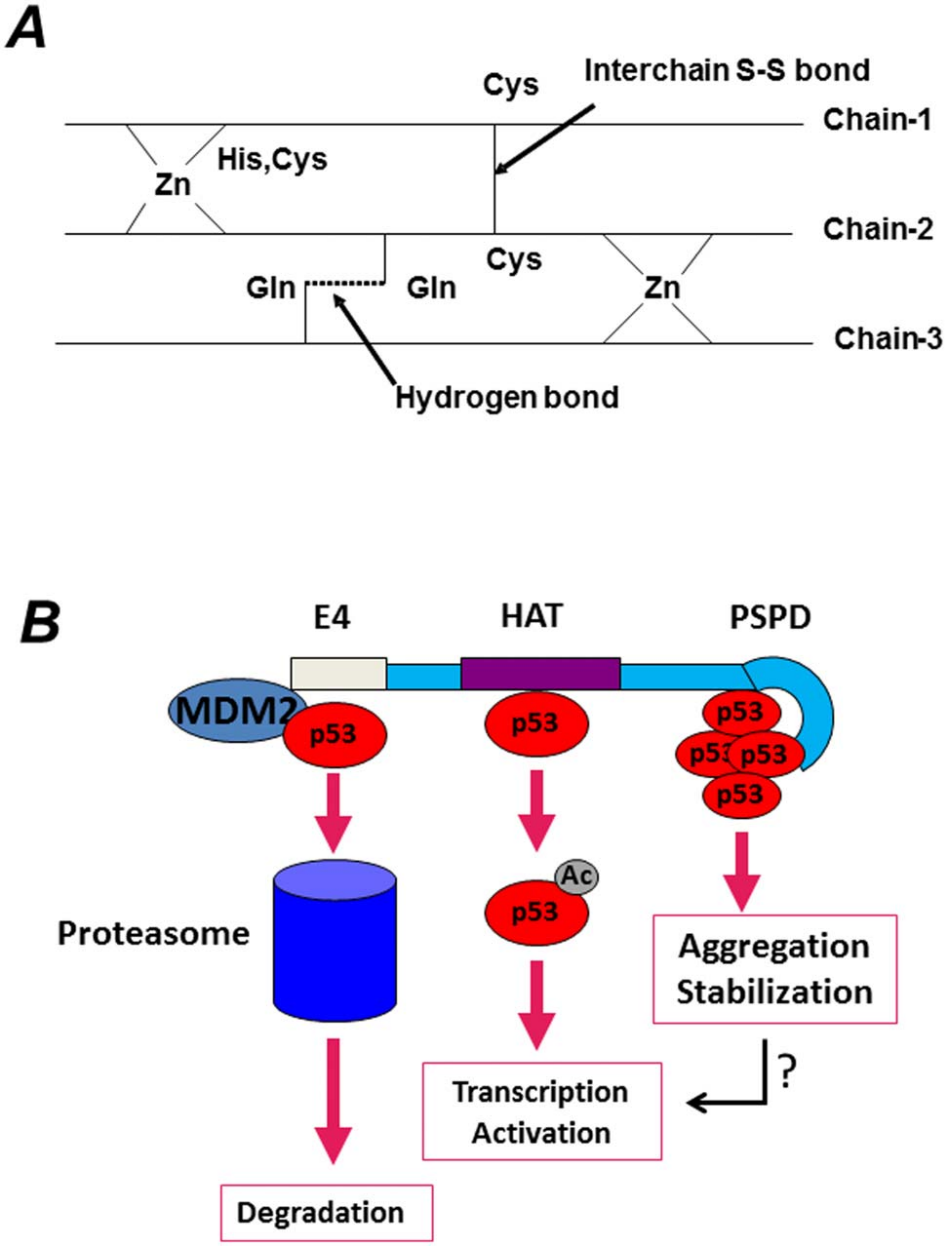
A. Proposed model for aggregation of the TAZ2 domain depending upon the concentration of zinc. See also text for explanation. The 1H-15N HSQC spectrum of the TAZ2 apoprotein at low concentration of zinc is poorly dispersed and has narrow line-widths. In low concentration of zinc, the cysteines residues in the fingers will be exposed to solvent in order to avoid intramolecular disulfide bridges. These exposed cysteines with histidines from two chains could interact with zinc and form interchain aggregates. Additional interchain stability could be obtained by forming interchain disulfide bridges between unligated cysteines residues as well as by forming two interchain hydrogen bonds among the side chains of glutamine and asparagine residues which are present throughout the sequence. A schematic representation of intermolecular aggregation is shown. **B.** Based on data presented here, and in keeping with previous studies that implicated p300 as an E4-ubiquitin ligase, as well as with evidence that p53 is activated by p300 when acetylated, we envision that p300 plays versatile and multiple effects on p53 activity. p53 interacts with at least three sites on p300, as indicated in the Figure, and each interaction site leads to different outcomes. While the interaction with the N-terminus serves to promote degradation of natively folded forms of p53 and to maintain low p53 levels in normal cells, the p300 PSPD brings p53 into cellular aggregates.

What are then, the physiological functions of these aggregation prone domains in p300 and CBP, why do transcription activators and co-repressors contain such domains, and why such domains aggregate in some contexts and not in others? Aside from post-translational modifications and protein-protein interactions, several studies have demonstrated the importance of metal ions in protein folding and aggregation, with zinc playing a very important role in this respect [52], [53]. The three-dimensional NMR structure of several portions of p300 or CBP has been determined. The NMR structure of the TAZ2 zinc-finger, for example, has shown that the spectrum of this domain at low concentration of zinc is poorly dispersed and has narrow line-widths, an indication that it is unfolded [54]. By contrast, the spectrum in the presence of three equivalents of zinc is typical of a well-folded protein. At low concentration of zinc, the cysteine residues are exposed to solvent in order to avoid intra-molecular disulfide bridges. In the model shown in **Figure 9A**, we propose that these exposed cysteine and histidine residues could form inter-chain aggregates stabilized by disulfide bridges between unligated cysteine residues as well as by inter-chain hydrogen bonds among the side chains of glutamine and asparagine residues, which are present throughout the CH3 and PSPD sequence. In fact, a total of 22% Q/N is found in the C-terminus of p300, and 17% are present in TAZ2 finger alone, an amount lower than typical prions, but certainly highly unusual compared to other zinc fingers. These intra-molecular interactions could contribute to aggregation and stabilize self- and hetero- aggregates depending upon zinc availability. It is therefore attractive to speculate that in the context of transcription, changes in the aggregation state of this region and its misfolding might expand the promiscuity of p300’s binding repertoire, leading to local aggregation on chromatin of factors required for activation or repression. In the cytoplasm, this domain might be involved in the interaction with various misfolded and ubiquitinated proteins and in their segregation into aggregates.

Thus, an important question for further studies will be to determine the molecular mechanisms that regulate the sub-cellular partitioning of p300. In keeping with the existence of an alternatively spliced variant of p300 encompassing the PSPD, and given that the p300 N-terminus contains the nuclear localization signal [55], alternative splicing could at least in part dictate p300’s subcellular distribution and also its tendency to aggregate in physiological or in pathological conditions.

### A Dual Role of p300 in Regulation of the Activity of p53

We have further shown that endogenous p53 and p300 colocalize in cytoplasmic inclusions and in aggresomes. Indeed, these inclusions contain ubiquitin, the 20S subunit of the proteasome and also cytoskeletal proteins such as vimentin. We have also shown that the PSPD of p300 is sufficient for inducing the formation of aggregates and to relocalize p53 in such structures. Interestingly, the CH3 within the PSPD domain contains, in addition to a p53-binding site, also a domain of interaction for the viral oncoproteins SV40 Tag and Adenoviral E1a, and we observed that in some cells expressing these viral proteins, such as in Hek293 cells, aggregates containing p53 and p300 form spontaneously, in the absence of proteasome inhibitors. Thus, it is possible that viral oncogenic pathways exploit this domain of p300 to mimic a misfolded protein response, and to seclude p53 into cytoplasmic aggregates thereby rendering it inactive. This novel activity of p300 that can be potentially relevant to oncogenic transformation.

The N-terminal region of p300 has been shown to possess E4, ubiquitin-chain elongating activity that induces poly-ubiquitination and degradation of p53 [19]. Indeed, the fact that p300 functions to destabilize wild-type p53 in certain conditions is supported by multiple lines of evidence, including demonstration that p300 enhances MDM2-mediated destruction of p53 [19], [20]. Thus, p300 might play a dual role in the stability of p53 (illustrated in **Figure 9B**). Specifically, on one side, p300 might promote p53 degradation via its N-terminal activities, on the other side, the C-terminal region of p300 might lead to p53 stabilization. Thus, the regulated action of different regions of p300 might dictate whether p53 should or should not be destined for disruption, depending upon its configuration, cellular context and post-translational modifications. Importantly, at least three interaction sites for p53 have been identified by us and by other laboratoties, one in the N-terminus, a second in the acetyl-transferase domain, and a third in the C-terminus of p300 [16]. Thus, does p53 differentially interacts with these p300’s domains in a signal or cell type-dependent manner? Further studies should address this question.

### p300 as an Integrator of Cellular Responses to Misfolded Protein Stress

We have further shown that p300 acts as a protective survival factor in the misfolded protein response. Thus, consistent with data from other groups [7], [8], our own data imply that aggresomes have a pro-survival and chemo-protective effect. It is likely that the sensitivity of cells to misfolded protein stress is determined by the integration of p300 multiple activities. Particularly, the positioning onto aggresomes of p300, a protein also endowed with transcriptional functions, might represent a strategy through which cells transduce stress signals arising from accumulation of misfolded proteins into proper adjustments of the cell cycle, a view consistent with results shown here. Additionally, given that proteasome inhibitors and drugs that affect protein-triage decisions have enter clinical testing in cancer and neurdegeneration [15], [55], our data may foster the development of novel molecules that, by targeting different p300 domains, affect life and death decisions in various pathogenic conditions.

## Methods

### Tissue Culture, Cell Treatments and Reagents

H1299 lung carcinoma cells, H1299 lung cancer cell line, A549 human lung epithelial cells, Hek293 human embryonic kidney cells, and COS7 transformed African Green Monkey kidney fibroblasts were obtained from ATCC or from the Tissue Culture Core Facility of Georgetown University. H1299 cells expressing wild-type p53, or p53 mutants at position R175H or G245A have been described previously [56], [57]. Cells were grown in Dulbecco’s modified Eagles’s Medium (DMEM) supplemented with 10% (v/v) fetal bovine serum, L-glutamine (5 mM), penicillin (100 U/ml), and streptomycine (100 µg/ml). Vectors for the tetracycline inducible system were purchased from Invitrogen and generation of cells lines was achieved accordingly to manufacturer instructions. Transfections were performed by using Lipofectamine (Invitrogen) or FuGene 6 (Roche) reagents.

#### RT-PCR

The primers for detection of the p300 alternatively spliced product were designed based on the sequences of the alternative exon provided by theAceview database and were as follows: Forward: 5′- ATGACAGAGCGAGGCCCTGTCT-3′; Reverse: 5′-CCATTGGTTTTCCGTTTGCAACCCT-3′. RT-PCR was performed with QIAGEN OneStep RT-PCR Kit.

### Immuno-blotting and Immuno-precipitations

For immuno-blotting cells were extracted in TBS Buffer (1%Triton X-100; 50 mM Tris-HCl pH 7.4; 150 mM NaCl; 1 mM EDTA). Cells were extracted in Buffer A (20% Glycerol; 40 mM Tris HCl pH 7.9; 0.4 mM EDTA; 0.2% Tween 20; 100 mM KCl), supplemented with protease inhibitors. Protein extracts were combined with the indicated antibody, precipitated with immobilized protein A beads (Pierce), and subjected to SDS-PAGE, followed by transfering to PVDF membranes (Milipore). Chemiluminescence was performed with the WestPico system (Pierce). The primary antibodies used for immunoblotting were: p300 [N15 and C-20, Santa Cruz Biotechnology (SCBT)]; α-tubulin (B-5-12, Sigma); FLAG (M2, Sigma); GRP78 (C20, SCBT); dynein, intermediate chain (74-1, SCBT); ubiquitin (P4D1, SCBT and 13-1600, Zymed); p53: [Ab-1 (pAb421), Ab-3 (pAb240) and Ab-6, (DO-1), Calbiochem; FL393, SCBT); hsp70 (W27, SCBT); HDAC6 (H-300 polyclonal rabbit; and D11 monoclonal mouse, SCBT).

### Recombinant Plasmids

For the generation of RFP-fusion proteins the p300 target sequence was PCR amplified with primers that incorporated an XhoI and KpnI restriction site into the 5′ and 3′ end of each amplicon, respectively. The resulting amplicons were then digested with XhoI and KpnI and cloned in frame with the pDsRed fluorescent protein in the pDsRed-Express-C1 vector (BD Biosciences). The primers employed were: RFP-CH3 (p300 sequence 1514–1922) 5′-aattgctcgaggcgaagaaagcattaaggaactgg-3′ and 5′-aattcggtacctgggggccctggaagg-3′; RFP-ZZ (p300 sequence 1620–1730) 5-aattgctcgaggcccctgcgatctgatggatg-3′ and 5′-aattcggtaccagaatcgcctgggctctgg-3′; RFP-TAZ2 (p300 sequence 1710–1820) 5′-aattgctcgaggccttggcttagatgatgagag-3′ and 5′-aattcggtaccgtgctgcagctgttgctgc-3′.

### Indirect Immuno-fluorescence

Cells were plated on glass cover slips, treated with 5 uM of MG132 for 16 hr or vehicle (DMSO), then fixed with 4% paraformaldehyde in PBS and permeabilized with 0.1% Triton X-100 in PBS for 10 min, followed by incubation with blocking solution (1% BSA in PBS). All antibodies were diluted in blocking solution. The antibodies employed for IF were: p300 (N15 or C20, SCBT); Vimentin (monoclonal-V9, polyclonal-H84, SCBT); p53 (monoclonal mix of Ab1 and Ab6, Calbiochem); Ubiquitin (monoclonal mix of P4D1 and 13–1600, SCBT and Zymed, respectively); GRP78 (C20, SCBT). After incubation with primary antibodies cells were incubated with the appropriate secondary antibodies: donkey anti-mouse or anti-rabbit Alexa488 and Alexa566 fluoro-conjugated antibodies (Molecular Probes, Invitrogen). The cover slips were washed with PBS, post-fixed with 4% parformaldehyde and nuclei were stained with DAPI and then mounted on slides with ProLong Gold anti-fade reagent (Invitrogen). Images were captured with an Axiovert 200 M fluorescence microscope (Zeiss), equipped with AxioCam MRm camera. The Z-stack images data obtained with 40x, 63x or 100x oil immersion objectives were processed and subjected to deconvolution with the Axio Vs40 software (Zeiss).

### Small Interfering RNAs

All the siRNA employed in this study were synthesized by Ambion. **CBP:** Sense: GGAAUAGGAAAUGUGAGCGtt; Antisense: CGCUCACAUUUCCUAUUCCtg. **p300:** Sense: AACCCCUCCUCUUCAGCACCA; antisense: UGGUGCUGAAGAGGAGGGGUU. For siRNA treatments, proliferating cells were treated with each siRNA (50–100 nM) reagent in serum-reduced Opti-MEM medium using Lipofectamine reagent (Invitrogen). Cells were incubated in the presence of siRNA for at least 72 hours after which time MG132 (Calbiochem) was added.

### Human Brain Specimens

Paraffin imbedded postmortem human brain sections from Parkinson’s disease (PD) and non-PD control (normal aging) patients, were from Neuropathology Lab, Johns Hopkins University School of Medicine (Baltimore, MD) and New York Brain Bank (NYBB) at Columbia University (New York, NY).

### Immunohistochemistry of Human Brain Sections

Paraffin imbedded postmortem human brain sections were subject to de-paraffinization, antigen retrieval, and melanin bleaching prior to blocking and primary antibodies incubation. In brief, the sections were de-paraffinized at 60°C for 1 h followed by Xylene delipidation and decreasing ethanol concentrations (100%–70%) for re-hydration. Antigens were retrieved with sodium citrate buffer incubation at 95°C fro 20 min. Melanin deposition of the human midbrain was bleached by 30 min incubation in 0.2% potassium manganate (KMnO_4_) and followed by 2 min incubation in hydrobromic acid water (3∶1). The sections were blocked in 4% normal goat serum (NGS) in 1×PBS with 0.1% Tryton-X-100 at room temperature (RT) for 1 h. The dilutions of the primary antibodies were as follows: for p300 (1∶250; C20 from Santa Cruz); alpha-synuclein (1∶100; monoclonal, Abcam); HDAC6 (D11; 1∶100); anti-ubiquitin (1∶100; monoclonal mix). The antibodies were mixed and diluted in 2%NGS/0.02% NaN_3_/1×PBS, and sections were incubated at 4°C overnight. Following washes, the sections were incubated with mixture of secondary antibodies Alexa Fluor® 594 goat anti-rabbit IgG (1∶100) and Alexa Fluor® 488 goat anti-mouse IgG (1∶100) in 2%NGS/0.02% NaN_3_/1×PBS at RT for 2 h. Sections were washed in 1×PBS and cover glassed with ProLong Gold mounting media (Molecular Probe/Life technologies). Fluorescent-labeled human brain section images were acquired with Carl Zeiss AxioPlan 2 fluorescent microscope with AxioVision Rel.4.8 software.

### Compositional Profiling

To gain insight into the relationships between sequence and disorder, the amino acid compositions in different data sets were compared using an approach developed for visualizing the amino acid composition biases in IDPs. The fractional difference in composition between a given set of the analyzed cytoplasmic sequences, intrinsically disordered proteins, and a set of ordered proteins was calculated for each amino acid residue. The fractional difference is calculated as (C_s1_–C_s2_)/C_s2_ values, where C_s1_ is a content of a given residue in a protein of interest (p300 or its C-terminal domain) and C_s2_ is the corresponding value for the sample set of ordered proteins from PDB [29]. Positive and negative values indicate residues in a given set that have more and less order, respectively. Confidence intervals were estimated using per-protein bootstrapping with 1000 iterations.

### Disorder Prediction

The intrinsic disorder propensities of the analyzed proteins were evaluated by four different disorder predictors of the PONDR family. The first one is PONDR® VL-XT [58], which applies various compositional probabilities and hydrophobic measures of amino acid as the input features of artificial neural networks for the prediction. Although it is no longer the most accurate predictor, it is very sensitive to the local compositional biases. Hence, it is capable of identifying potential molecular interaction motifs. The second and third predictors are PONDR® VSL2B [59], which is suitable for accurate evaluation of short and long disordered regions, and PONDR® VL3 [60], which is suitable for finding long disordered regions. The last tool is a meta-predictor PONDR-FIT that combines six individual predictors, which are PONDR® VL-XT, VSL2, VL3, FoldIndex, IUPred, and TopIDP. This meta-predictor is moderately more accurate than each of the component predictors [61].

### Molecular Recognition Feature (MoRF) and ANCHOR Analysis

Being defined as a short order-prone motif within a long disordered region and being able to undergo disorder-to-order transition during the binding to a specific partner, Molecular Recognition Feature (MoRF) usually has much higher content of aliphatic and aromatic amino acids than disordered regions in general. Due to these peculiarities, MoRF regions are frequently observed as sharp dips in the corresponding plots representing per-residue distribution of PONDR® VL-XT disorder scores. Hence, based on the PONDR® VL-XT prediction and a number of other attributes, the MoRF regions can be identified [30], [31]. In addition to MoRF identifiers, potential binding sites in disordered regions can be identified by the ANCHOR algorithm [32]. This approach relies on the pairwise energy estimation approach developed for the general disorder prediction method IUPred, being based on the hypothesis that long regions of disorder contain localized potential binding sites that cannot form enough favorable intrachain interactions to fold on their own, but are likely to gain stabilizing energy by interacting with a globular protein partner. Here we are using the term ANCHOR-indicated binding site (AIBS) to identify a region of a protein suggested by the ANCHOR algorithm to have significant potential to be a binding site for an appropriate but typically unidentified partner protein.

## Supporting Information

Figure S1 **A.** H1299 cells were mock treated (A) or treated with 5 µM MG132 for 16 hours (BC) and cells were stained with a monoclonal mix of anti-ubiquitin, anti-p300, or anti-vimentin monoclonal as indicated in each panel. The arrows indicate the position of representative aggresomes. **B.** Human embryonic kidney carcinoma cells Hek293 cells were stained for p300 (green), and vimentin, as indicated at the top of each panel.
(TIF)

Figure S2 **Detection of p300 in cytoplasmic inclusions in the absence of MG132 treatment. A.** Untreated or MG132 treated Cos-7 cells grown on coverslips were stained with anti-vimentin and anti-CBP (C22) antibodies.
(TIF)

Figure S3 **The p300-PSPD brings TAU in cytoplasmic aggregates. A.** Co-localization of endogenous p300 and TAU. Cos-7 cells were transfected with the vector expressing TAU and 24 hours after transfection cells were stained with the polyclonal antibody directed against p300 (C20, red, *1*) and with a monoclonal antibody recognizing TAU (green, *2*). In a parallel set of experiments, cells were co-transfected with the RFP-PSPD and TAU expressing vectors (**B**), and stained as described in A. The white rectangle in panels A and B demarks areas of co-localization of p300 with TAU tangles (3) or with aggregates (6). These areas are enlarged in panel **D**. **C**. Quantification of experiments shown in panel B. Cells with no aggregates (gray bars), and cells with aggregates (black bars) were counted in the presence (+) or absence (−) of the p300-PSPD. Numbers represents average percentage of cells. **E-F. p300 and p53 co-localize in cytoplasmic aggregates.** Hek293 cells were plated on glass coverslips and stained with the p53 monoclonal antibody (red) and the p300 polyclonal antibody (green) (panel **E**); or with the p53 monoclonal antibody and a polyclonal antibody recognizing the 20S subunit of the proteasome (**F**). Arrows indicate inclusion bodies in cells where p53, p300 and the 20S proteasome co-localize. Approximately 60% of Hek293 cells display these inclusions.
(TIF)

Figure S4 **The p300-PSPD brings p53 and p21/WAF in cytoplasmic aggregates. A–D.** H1299 cells expressing tetracycline-inducible p53 protein [56], were transfected with different p300 mutants previously described in Figure 3, specifically with the p300-Δ30 (**A**), p300-N (**B**) and p300-PSPD (**C–D**). Twenty four hours after induction of p53 via tetracycline addition, a set of cells transfected with the p300-PSPD were treated with MG132 (**D**), then cells were fixed and stained for p53 and p300. In panel C and D, aggregates forming in cells expressing both p300 and p53 are indicated by white arrows. Red arrows indicate cells expressing p53 but not the p300-PSPD.
(TIF)

Figure S5 **The p300-PSPD promotes p53 mutant aggregation.** H1299 cell lines expressing p53G245A (panel A and B), or p53R175H (panel C) [57], were seeded on glass cover-slips and were transfected with RFP or with RFP-PSPD. After transfection cells were stained with the anti-p53 antibody (FL393). Note the nuclear pattern of localization of the p53 mutants in cells not expressing the p300-PSPD. The white rectangles mark cells co-expressing p300 and p53 and containing aggregates, which are enlarged at the right of the merge panel.
(TIF)

Table S1 **Similarity between the p300 region comprised between amino acid 1688 to 1214 and various proteins containing prion-like domains in C.** ***elegans*** **(** ***pqn*** **and** ***Abu*** **family), and other species.** All sequences found had a blast e-value above 0.001.
(DOCX)

Movie S1 **Dynamics of aggresomes in the presence of RFP-p300-PSPD.** Cells were transfected with the vector expressing RFP-p300-PSPD, plated onto Lab-Tek II Chambered coverglasses System dish, and then treated with 5 µM MG132. The dishes were placed in a temperature-controlled stage at 37°C on an inverted microscope (Nikon Eclipse TE300) in a 5% CO_2_ atmosphere, and subjected to time-lapse video microscopy. Images were collected using a 40x lens, with a filter set for DsRED (excitation 545 nm, dichroic 570 nm, emission 620 nm), and a Hamamatsu Orca-ER, monochrome cooled CCD camera. Images were taken at 15 min intervals for 16 h with MetaMorph Imaging system, and were then assembled into QuickTime movies.
(WMV)
