# Supplementary figures and images for: An Intrinsically Disordered Region of the Acetyltransferase p300 with Similarity to Prion-Like Domains Plays a Role in Aggregation
Source: PLoS One. 2012 Nov 1;7(11):e48243. doi: 10.1371/journal.pone.0048243 (PMC3486812; doi:10.1371/journal.pone.0048243)

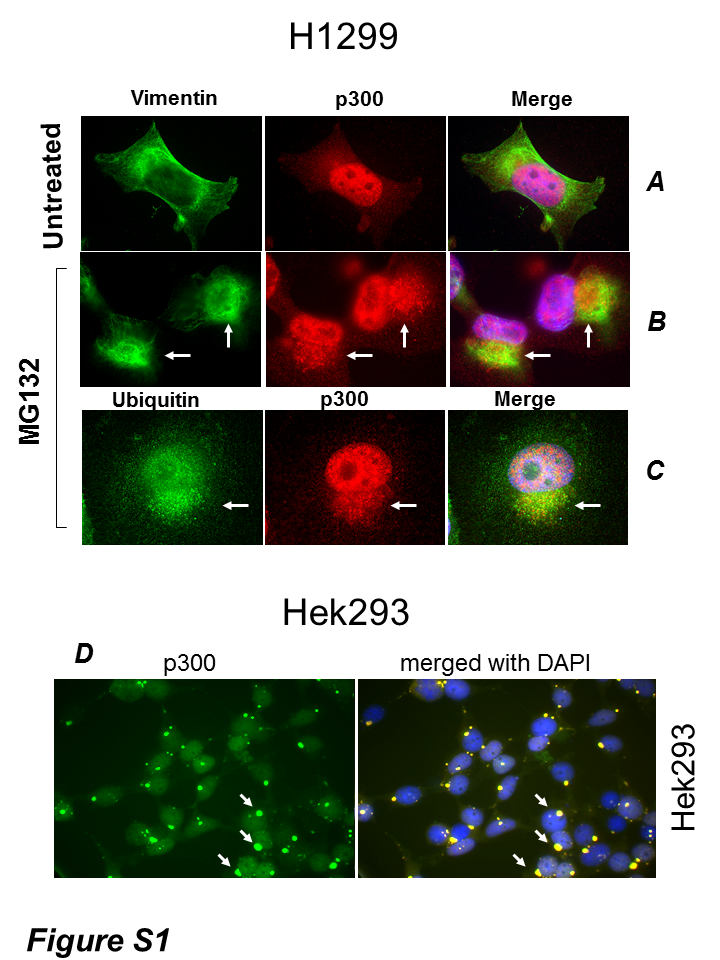

Supplement: Figure S1 — A. H1299 cells were mock treated (A) or treated with 5 µM MG132 for 16 hours (BC) and cells were stained with a monoclonal mix of anti-ubiquitin, anti-p300, or anti-vimentin monoclonal as indicated in each panel. The arrows indicate the position of representative aggresomes. B. Human embryonic kidney carcinoma cells Hek293 cells were stained for p300 (green), and vimentin, as indicated at the top of each panel. (TIF) [file pone.0048243.s001.tif]

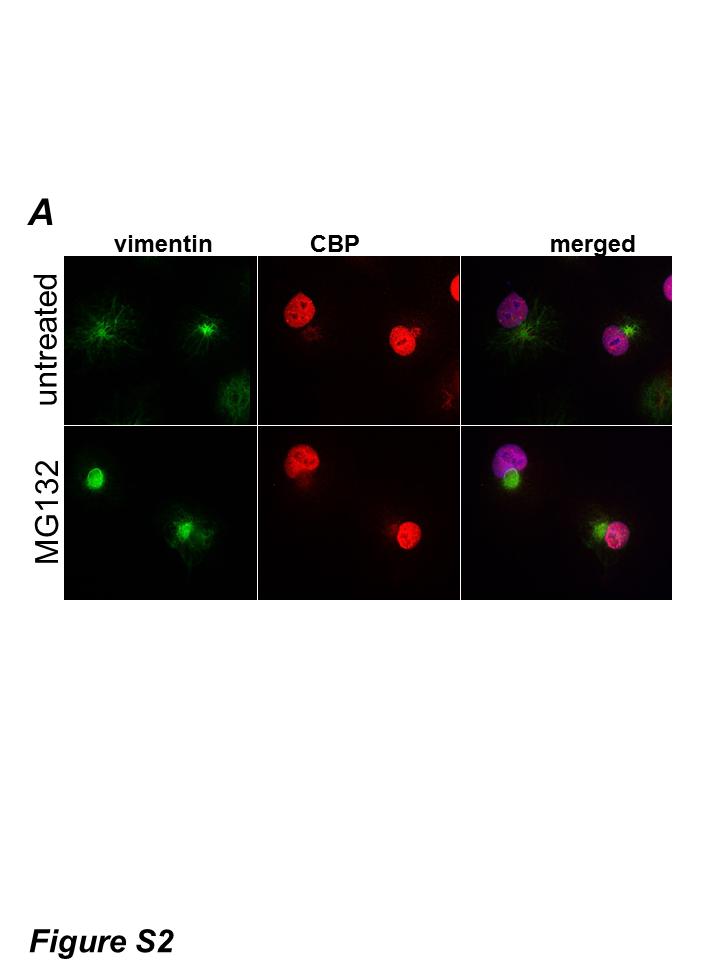

Supplement: Figure S2 — Detection of p300 in cytoplasmic inclusions in the absence of MG132 treatment. A. Untreated or MG132 treated Cos-7 cells grown on coverslips were stained with anti-vimentin and anti-CBP (C22) antibodies. (TIF) [file pone.0048243.s002.tif]

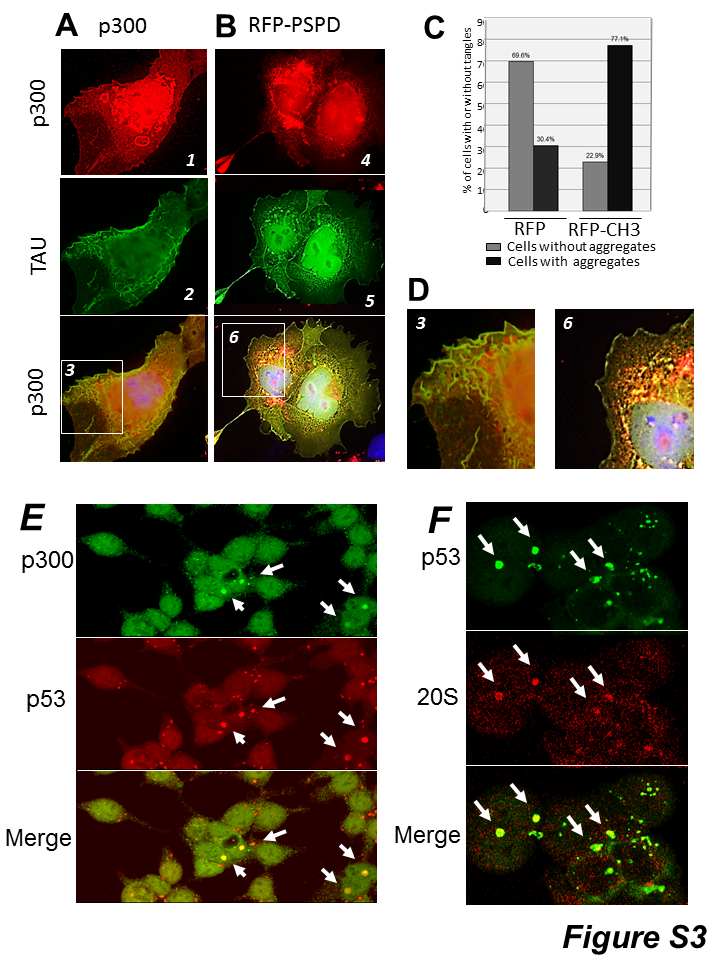

Supplement: Figure S3 — The p300-PSPD brings TAU in cytoplasmic aggregates. A. Co-localization of endogenous p300 and TAU. Cos-7 cells were transfected with the vector expressing TAU and 24 hours after transfection cells were stained with the polyclonal antibody directed against p300 (C20, red, 1) and with a monoclonal antibody recognizing TAU (green, 2). In a parallel set of experiments, cells were co-transfected with the RFP-PSPD and TAU expressing vectors (B), and stained as described in A. The white rectangle in panels A and B demarks areas of co-localization of p300 with TAU tangles (3) or with aggregates (6). These areas are enlarged in panel D. C. Quantification of experiments shown in panel B. Cells with no aggregates (gray bars), and cells with aggregates (black bars) were counted in the presence (+) or absence (−) of the p300-PSPD. Numbers represents average percentage of cells. E-F. p300 and p53 co-localize in cytoplasmic aggregates. Hek293 cells were plated on glass coverslips and stained with the p53 monoclonal antibody (red) and the p300 polyclonal antibody (green) (panel E); or with the p53 monoclonal antibody and a polyclonal antibody recognizing the 20S subunit of the proteasome (F). Arrows indicate inclusion bodies in cells where p53, p300 and the 20S proteasome co-localize. Approximately 60% of Hek293 cells display these inclusions. (TIF) [file pone.0048243.s003.tif]

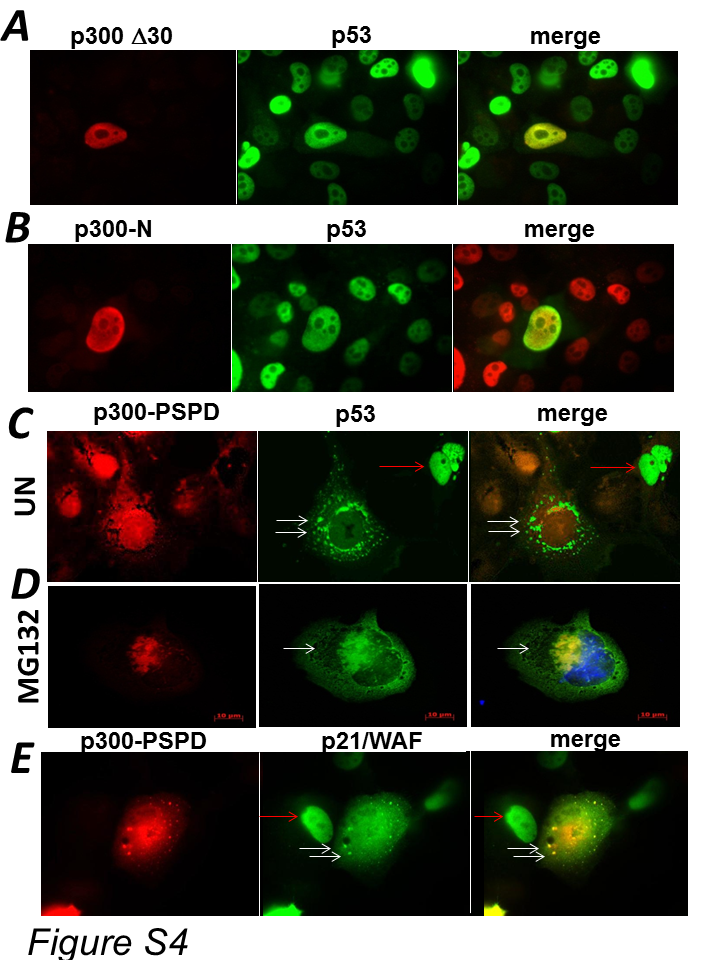

Supplement: Figure S4 — The p300-PSPD brings p53 and p21/WAF in cytoplasmic aggregates. A–D. H1299 cells expressing tetracycline-inducible p53 protein [56], were transfected with different p300 mutants previously described in Figure 3, specifically with the p300-Δ30 (A), p300-N (B) and p300-PSPD (C–D). Twenty four hours after induction of p53 via tetracycline addition, a set of cells transfected with the p300-PSPD were treated with MG132 (D), then cells were fixed and stained for p53 and p300. In panel C and D, aggregates forming in cells expressing both p300 and p53 are indicated by white arrows. Red arrows indicate cells expressing p53 but not the p300-PSPD. (TIF) [file pone.0048243.s004.tif]

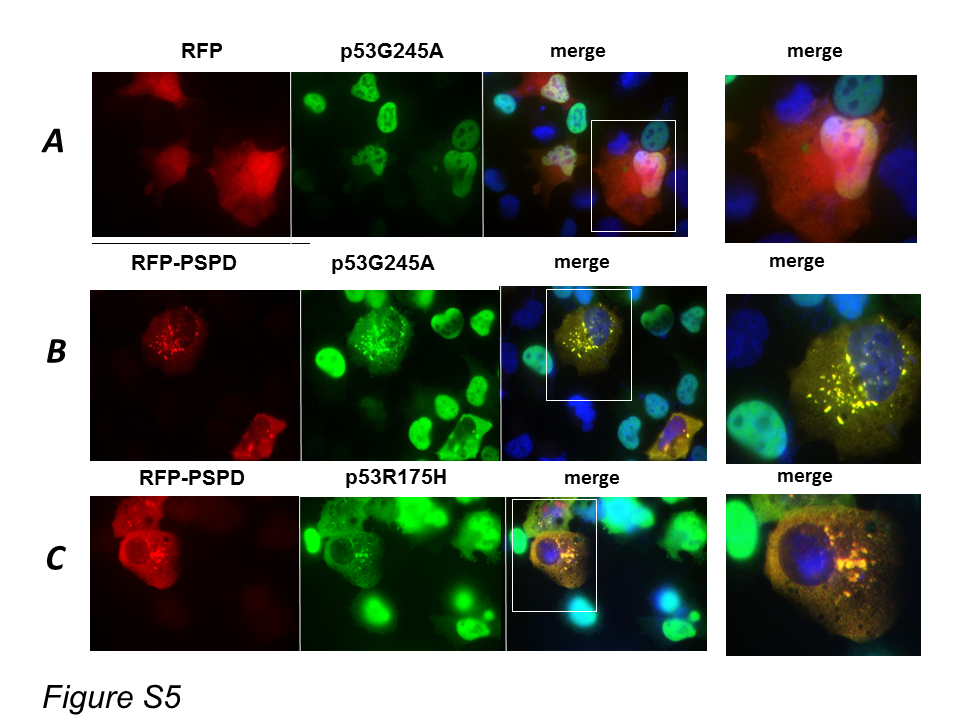

Supplement: Figure S5 — The p300-PSPD promotes p53 mutant aggregation. H1299 cell lines expressing p53G245A (panel A and B), or p53R175H (panel C) [57], were seeded on glass cover-slips and were transfected with RFP or with RFP-PSPD. After transfection cells were stained with the anti-p53 antibody (FL393). Note the nuclear pattern of localization of the p53 mutants in cells not expressing the p300-PSPD. The white rectangles mark cells co-expressing p300 and p53 and containing aggregates, which are enlarged at the right of the merge panel. (TIF) [file pone.0048243.s005.tif]
