# Supplementary material for: An Intrinsically Disordered Region of the Acetyltransferase p300 with Similarity to Prion-Like Domains Plays a Role in Aggregation
Source: PLoS One. 2012 Nov 1;7(11):e48243. doi: 10.1371/journal.pone.0048243 (PMC3486812; doi:10.1371/journal.pone.0048243)
Supplement: Table S1 — Similarity between the p300 region comprised between amino acid 1688 to 1214 and various proteins containing prion-like domains in C. elegans ( pqn and Abu family), and other species. All sequences found had a blast e-value above 0.001. (DOCX) [file pone.0048243.s006.docx]

**Table S1.** Results of the BLAST search for the p300 PSPD domain. The sequence of the C-terminal part of PSPD (residues 1688-2414) was used in this analysis

1688-DYDLCITCYNTKNHDHKMEKLGLGLDDESNNQQAAATQSPGDSRRLSIQRCIQSLVHACQCRNANCSLPSCQKMKRVVQHTKGCKRKTNGGCPICKQLIALCCYHAKHCQENKCPVPFCLNIKQKLRQQQLQHRLQQAQMLRRRMASMQRTGVVGQQQGLPSPTPATPTTPTGQQPTTPQTPQPTSQPQPTPPNSMPPYLPRTQAAGPVSQGKAAGQVTPPTPPQTAQPPLPGPPPAAVEMAMQIQRAAETQRQMAHVQIFQRPIQHQMPPMTPMAPMGMNPPPMTRGPSGHLEPGMGPTGMQQQPPWSQGGLPQPQQLQSGMPRPAMMSVAQHGQPLNMAPQPGLGQVGISPLKPGTVSQQALQNLLRTLRSPSSPLQQQQVLSILHANPQLLAAFIKQRAAKYANSNPQPIPGQPGMPQGQPGLQPPTMPGQQGVHSNPAMQNMNPMQAGVQRAGLPQQQPQQQLQPPMGGMSPQAQQMNMNHNTMPSQFRDILRRQQMMQQQQQQGAGPGIGPGMANHNQFQQPQGVGYPPQQQQRMQHHMQQMQQGNMGQIGQLPQALGAEAGASLQAYQQRLLQQQMGSPVQPNPMSPQQHMLPNQAQSPHLQGQQIPNSLSNQVRSPQPVPSPRPQSQPPHSSPSPRMQPQPSPHHVSPQTSSPHPGLVAAQANPMEQGHFASPDQNSMLSQLASNPGMANLHGASATDLGLSTDNSDLNSNLSQSTLDIH-2414

**1.>**[ref|NP_493687.2|](http://www.ncbi.nlm.nih.gov/protein/71998210?report=genbank&log$=protalign&blast_rank=3&RID=E9YHFVPV013) Prion-like-(Q/N-rich)-domain-bearing protein family member (pqn-85)

[Caenorhabditis elegans]

Length=2203

[GENE ID: 173410 pqn-85](http://www.ncbi.nlm.nih.gov/sites/entrez?db=gene&cmd=search&term=173410&RID=E9YHFVPV013&log$=geneexplicitprot&blast_rank=3) | Prion-like-(Q/N-rich)-domain-bearing protein

p300 361 QQALQNLLRTLRSPSSPLQQQQVLSILHANPQLLAAFIKQRAAKYANSN 409

+Q L L + +SP QQQQ + +L Q L F++ K A S+

Sbjct 1758 RQLTNTYLEILNAANSPQQQQQRILVL----QNLEMFLQCEEQKLAASH 1802

p300 120 LNIKQKLRQQQLQHRLQQAQML 141

L +Q+LR+Q + RL + + L

Sbjct 233 LEEQQRLREQMERERLAEIKRL 254

p300 533 PPQQQQRM 540

P QQQQR+

Sbjct 1774 PQQQQQRI 1781

p300 464 QQQLQPPMGGMSPQAQQMNMNHNTMPSQFRDILRRQQMMQQQQQQ 508

+Q+ MG Q + + + + +IL QQQQQ+

Sbjct 1737 RQKALTAMGHFCAQ-HSTYLTKRQLTNTYLEILNAANSPQQQQQR 1780

p300 476 PQAQQMNMN 484

PQAQ MN

Sbjct 107 PQAQHHQMN 115

p300 266 QHQMPPMT 273

QH +PP T

Sbjct 153 QHTIPPST 160

p300 158 QGLPSPTPATPT 169

QGL +P + PT

Sbjct 1861 QGLVTPGASIPT 1872

p300 648 PSPHHVSPQTSS 659

P P H P ++S

Sbjct 150 PIPQHTIPPSTS 161

p300 534 PQQQQRMQHHMQQMQ 548

PQQQQ+ +Q ++

Sbjct 1774 PQQQQQRILVLQNLE 1788

p300 447 NPMQAGVQRAGLPQQQPQQQ 466

N +Q + G P QP Q

Sbjct 4 NNLQNSLNGTGNPNFQPVQT 23

p300 566 AGASLQAYQQRLLQQQ 581

A S Q QQR+L Q

Sbjct 1770 AANSPQQQQQRILVLQ 1785

**2.** >[ref|NP_508659.1|](http://www.ncbi.nlm.nih.gov/protein/17567447?report=genbank&log$=protalign&blast_rank=2&RID=EW1ZFVM7013) Activated in Blocked Unfolded protein response family member


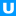

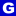

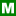


(abu-10) [Caenorhabditis elegans]

[gb|AAB52643.1|](http://www.ncbi.nlm.nih.gov/protein/1166616?report=genbank&log$=protalign&blast_rank=2&RID=EW1ZFVM7013) Activated in blocked unfolded protein response protein 10 [Caenorhabditis


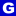


elegans] Length=375

[GENE ID: 185248 abu-10](http://www.ncbi.nlm.nih.gov/sites/entrez?db=gene&cmd=search&term=185248&RID=EW1ZFVM7013&log$=geneexplicitprot&blast_rank=2) | Activated in Blocked Unfolded protein response

[Caenorhabditis elegans] (10 or fewer PubMed links)

p300 265 IQHQMPPMTPMAPMGMNPPPMTRGPSGHLEPGM--GPTGMQQQPPWSQGGLPQPQQLQSG 322

Q Q AP+ NP P+ P + P QQ P QQ Q

Sbjct 241 CQQQAQSTCDCAPVQQNPCCQQAQPACDCAPVVQQNPCACQQAQPSCDCA----QQAQ-- 294

p300 323 MPRPAMMSVAQHGQPLN 339

P Q QP+N

Sbjct 295 ---PTFQVQVQQAQPIN 308

p300 390 NPQLLAAF---IKQRAAKYANSNPQPIPGQPGMPQGQPGLQPPTMPGQQGVHSNPAMQNM 446

NP + I+Q+ A+ + P+ P Q QP + Q P QN

Sbjct 151 NPSCVTCIQIEIRQQQAQQPACDCAPVQQNPCCQQAQPACDCAPVQQQPSCDCAPVQQNP 210

p300 447 NPMQA 451

QA

Sbjct 211 CCQQA 215

p300 586 VQPNPMSPQQHMLPNQAQSPHLQGQQIPNSLSNQVRSPQPVPSPRPQSQPPHSSPSPRMQ 645

VQ NP + QQ AQ QQ + QV+ QP+ P QP S

Sbjct 273 VQQNPCACQQ------AQPSCDCAQQAQPTFQVQVQQAQPINQCVPACQPACQSSCTASY 326

p300 646 PQPSPHHVSPQTSSPHPGLVAAQANPMEQGHFASPDQNSMLSQLASN 692

H S TS V ++G+ +Q L +N

Sbjct 327 TNQQSQHSSATTSCDANQCV------CQRGYVKCAEQTCCLRYRYTN 367

p300 412 PIPGQPGMPQGQPGLQPPTMPGQQGVHSNPAMQN 445

P+ P Q QP + Q P QN

Sbjct 205 PVQQNPCCQQAQPACDCAPVQQQPACDCAPVQQN 238

p300 206 AGPVSQGKAAGQVTPPT---PPQTAQPPLPGPP---PAAVEMAMQIQRAAETQRQMAHVQ 259

PV Q A Q P+ P QP P PA + Q A + Q+ + V

Sbjct 97 CAPVQQDPCACQQQQPSCDCAPVQQQPACDCQPQQQPACDCAPVAQQPACDCQQNPSCVT 156

p300 260 IFQRPIQHQMP--PMTPMAPMGMNPPPMTRGPSGHLEPGMGPTGMQQQP 306

Q I+ Q P AP+ NP P+ P +QQQP

Sbjct 157 CIQIEIRQQQAQQPACDCAPVQQNPCCQQAQPACDCAP------VQQQP 199

p300 169 TTPTGQQPTTPQTPQPTSQPQPT 191

P Q P Q QP+ P

Sbjct 97 CAPVQQDPCACQQQQPSCDCAPV 119

**3.** >[ref|NP_872210.1|](http://www.ncbi.nlm.nih.gov/protein/32567356?report=genbank&log$=protalign&blast_rank=3&RID=EW1ZFVM7013) Activated in Blocked Unfolded protein response family member

(abu-4) [Caenorhabditis elegans] Length=339

[GENE ID: 353458 abu-4](http://www.ncbi.nlm.nih.gov/sites/entrez?db=gene&cmd=search&term=353458&RID=EW1ZFVM7013&log$=geneexplicitprot&blast_rank=3) | Activated in Blocked Unfolded protein response

[Caenorhabditis elegans]

Score = 16.2 bits (30), Expect = 38, Method: Compositional matrix adjust.

Identities = 13/42 (31%), Positives = 18/42 (43%), Gaps = 5/42 (12%)

p300 429 PTMPGQQGVHSNP-----AMQNMNPMQAGVQRAGLPQQQPQQ 465

P+ QQ ++ P + QN P+Q A QQQ Q

Sbjct 39 PSCSCQQTTYAQPPQYSCSCQNTAPVQTSCSCAQPVQQQTYQ 80

p300 427 QPPTMPG-QQGVHSNPAMQNMNPM 449

QP MP + ++S QN +P

Sbjct 307 QPAQMPCMTESINSCSCQQNYSPC 330

p300 641 SPRMQPQPS 649

+PR+QP S

Sbjct 34 APRVQPSCS 42

p300 426 LQPPTMPG--QQGVHSNPAMQNMN 447

QP MP Q V P + ++N

Sbjct 128 CQPSCMPACKQSCVAPAPQIISLN 151

p300 172 TGQQPTTPQTPQPTSQPQPTPP 193

+ QQ T Q PQ + Q T P

Sbjct 42 SCQQTTYAQPPQYSCSCQNTAP 63

p300 67 SLPSCQKMKRVVQHTKGCKRK 87

+ SCQ+ + + CKRK

Sbjct 319 NSCSCQQNYSPCGNGQCCKRK 339

**4.>**[ref|NP_493679.1|](http://www.ncbi.nlm.nih.gov/protein/17535887?report=genbank&log$=protalign&blast_rank=4&RID=EW1ZFVM7013) Activated in Blocked Unfolded protein response family member


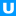

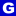

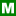


(abu-11) [Caenorhabditis elegans]

Length=395

[GENE ID: 173404 abu-11](http://www.ncbi.nlm.nih.gov/sites/entrez?db=gene&cmd=search&term=173404&RID=EW1ZFVM7013&log$=geneexplicitprot&blast_rank=4) | Activated in Blocked Unfolded protein response

[Caenorhabditis elegans] (10 or fewer PubMed links)

Score = 16.2 bits (30), Expect = 38, Method: Compositional matrix adjust.

Identities = 7/19 (37%), Positives = 10/19 (53%), Gaps = 0/19 (0%)

p300 193 PNSMPPYLPRTQAAGPVSQ 211

P ++P Y +Q A P Q

Sbjct 262 PGAIPSYQGASQTAAPTYQ 280

p300 180 QTPQPTSQP 188

QT PT QP

Sbjct 273 QTAAPTYQP 281

p300 641 SPRMQPQ 647

SP+ QPQ

Sbjct 356 SPQCQPQ 362

p300 523 QFQQPQGVG 531

Q QQP G

Sbjct 63 QAQQPSSCG 71

p300 437 VHSNPAMQNMNPMQA 451

+N A Q+ + QA

Sbjct 50 AQTNTAQQSCSCAQA 64

p300 179 PQTPQPTSQPQPT 191

P PQ QP T

Sbjct 40 PAAPQCNCQPAQT 52

**5.>**[ref|NP_502873.1|](http://www.ncbi.nlm.nih.gov/protein/17544298?report=genbank&log$=protalign&blast_rank=5&RID=EW1ZFVM7013) Activated in Blocked Unfolded protein response family member


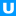

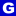

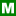


(abu-5) [Caenorhabditis elegans] Length=335

[GENE ID: 190870 abu-5](http://www.ncbi.nlm.nih.gov/sites/entrez?db=gene&cmd=search&term=190870&RID=EW1ZFVM7013&log$=geneexplicitprot&blast_rank=5) | Activated in Blocked Unfolded protein response

[Caenorhabditis elegans]

Score = 16.2 bits (30), Expect = 43, Method: Compositional matrix adjust.

Identities = 11/27 (41%), Positives = 13/27 (48%), Gaps = 1/27 (4%)

p300 427 QPPTMPG-QQGVHSNPAMQNMNPMQAG 452

QP MP Q V+S QN +P G

Sbjct 303 QPVQMPCLTQSVNSCSCQQNYSPCGNG 329

p300 429 PTMPGQQGVHSNP-----AMQNMNPMQAGVQRAGLPQQQPQQQL 467

P+ Q+ ++ P + QN P+Q A QP QQL

Sbjct 37 PSCSCQRTTYTQPQQYSCSCQNTAPVQTSCSCA-----QPVQQL 75

p300 574 QQRLLQQQMGSPVQPNPMSPQQHMLPNQAQS 604

QQ +PV P P+ Q +P QS

Sbjct 110 QQTCQTSSCYTPVAPAPVQCQPSCMPVCEQS 140

p300 400 QRAAKYANSNPQPIPGQPGM 419

Q ++ Y P P+ QP

Sbjct 114 QTSSCYTPVAPAPVQCQPSC 133

p300 57 HACQCRN 63

++C C+N

Sbjct 52 YSCSCQN 58

p300 414 PGQPGMPQGQPGLQP 428

P P Q QP P

Sbjct 121 PVAPAPVQCQPSCMP 135

p300 472 GGMSPQAQQMNMNHNTM 488

++P Q +++N +

Sbjct 140 SCVAPAPQVISLNLEVV 156

p300 26 DDESNNQQAAATQS 39

+ +S+ QQ T S

Sbjct 104 NCQSSCQQTCQTSS 117

**6**. >[ref|NP_508265.1|](http://www.ncbi.nlm.nih.gov/protein/17567159?report=genbank&log$=protalign&blast_rank=6&RID=EW1ZFVM7013) Activated in Blocked Unfolded protein response family member


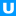

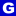

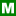


(abu-2) [Caenorhabditis elegans] Length=572

[GENE ID: 184695 abu-2](http://www.ncbi.nlm.nih.gov/sites/entrez?db=gene&cmd=search&term=184695&RID=EW1ZFVM7013&log$=geneexplicitprot&blast_rank=6) | Activated in Blocked Unfolded protein response

[Caenorhabditis elegans]

Score = 16.2 bits (30), Expect = 50, Method: Compositional matrix adjust.

Identities = 13/43 (30%), Positives = 18/43 (42%), Gaps = 0/43 (0%)

p300 420 PQGQPGLQPPTMPGQQGVHSNPAMQNMNPMQAGVQRAGLPQQQ 462

P+ QP P Q ++ + QN P+Q A QQQ

Sbjct 33 PKVQPSCSCQQTPYTQPQETSCSCQNTAPVQTSCSCAQPVQQQ 75

p300 421 QGQPGLQPPTMPG-QQGVHSNPAMQNMNPM 449

Q QP MP Q +S QN +P

Sbjct 534 QSCSAAQPAQMPCITQSANSCSCQQNYSPC 563

p300 93 PICKQLIALCCY 104

P+C+Q A CY

Sbjct 424 PVCEQQCAPQCY 435

p300 93 PICKQLIA 100

P+C+Q A

Sbjct 394 PVCEQQCA 401

p300 207 GPVSQGKAAGQVTPPTPP 224

PV + + A Q P+ P

Sbjct 423 APVCEQQCAPQCYQPSAP 440

p300 184 PTSQPQPT 191

P +QPQ T

Sbjc 45 PYTQPQET 52

p300 67 SLPSCQKMKRVVQHTKGCKRK 87

+ SCQ+ + + CKR+

Sbjct 552 NSCSCQQNYSPCGNGQCCKRR 572

**7.>**[ref|XP_001232979.1|](http://www.ncbi.nlm.nih.gov/protein/118102149?report=genbank&log$=protalign&blast_rank=7&RID=EW1ZFVM7013) PREDICTED: similar to Activated in blocked unfolded protein response


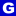


protein 2 [Gallus gallus] Length=463

[GENE ID: 769688 KRTAP9-1](http://www.ncbi.nlm.nih.gov/sites/entrez?db=gene&cmd=search&term=769688&RID=EW1ZFVM7013&log$=geneexplicitprot&blast_rank=7) | keratin associated protein 9-1 [Gallus gallus]

Score = 15.8 bits (29), Expect = 60, Method: Compositional matrix adjust.

Identities = 8/23 (35%), Positives = 11/23 (48%), Gaps = 1/23 (4%)

p300 308 WSQGGLPQPQQLQSGMPRPAMMS 330

W Q L PQ + +PR +S

Sbjct 4 W-QSELCTPQYVTPCVPRQRFLS 25

p300 578 LQQQMGSPVQPNPMSPQQHML 598

Q ++ +P P P+Q L

Sbjct 4 WQSELCTPQYVTPCVPRQRFL 24

p300 435 QGVHSNPAMQNMNPMQ 450

QG+ P + P Q

Sbjct 76 QGMSQEPCVTKCMPQQ 91

p300 151 TGVVGQQQGLPSPTP---ATPTTPTGQQPTTPQTPQ 183

T + QQQ P AT P QQ T PQ

Sbjct 363 TKCIPQQQCATKCAPQQCATKCIPQQQQCATKCVPQ 398

p300 157 QQGLPSPTPATPTTP 171

Q L +P TP P

Sbjct 5 QSELCTPQYVTPCVP 19

p300 305 QPPWSQGGL 313

Q P+ GG+

Sbjct 438 QQPYQSGGV 446

p300 570 LQAYQQRLLQQQMGSPVQP------NPMSPQQHM 597

+ ++Q L Q +P P + SPQQ +

Sbjct 1 MHSWQSELCTPQYVTPCVPRQRFLSSSFSPQQCV 34

p300 301 GMQQQP 306

GM Q+P

Sbjct 77 GMSQEP 82

p300 429 PTMPGQQGVHSNPAMQNMNPMQAGVQRAGLPQQQ 462

P +P Q+ + S + +P Q Q +P+QQ

Sbjct 16 PCVPRQRFLSS-----SFSPQQCVTQ--CIPRQQ 42

p300 579 QQQMGSPVQPNPMS----PQQH 596

QQQ + P + PQQH

Sbjct 388 QQQCATKCVPQQCATKGIPQQH 409

p300 457 GLPQQQ 462

+PQQQ

Sbjct 202 CIPQQQ 207

p300 457 GLPQQQPQQQLQPPMGGMSPQAQQMNMNHNTMPSQF 492

+PQQQ + P QQ +P Q

Sbjct 365 CIPQQQCATKCAPQQCATKCIPQQQQCATKCVPQQC 400

p300 506 QQQGAGPGIGPGM 518

QQQ A + +

Sbjct 110 QQQCAARCVTTCI 122

p300 305 QPPWSQGGLPQPQQLQSGMPRPAMMSVAQHGQ 336

Q P+ G+ Q +P+ + + A H Q

Sbjct 124 QQPFLTKGIRQQHSATVCIPQHCVTTYAPHEQ 155

p300 614 NSLSNQVRSPQPVPSPRPQSQPPHSSPSPR 643

+S +++ +PQ V P+ + SS SP+

Sbjct 2 HSWQSELCTPQYVTPCVPRQRFLSSSFSPQ 31

p300 275 MAPMGMNPPPM 285

A GM+ P

Sbjct 73 CAAQGMSQEPC 8

**8.>**[gb|AAN88028.1|](http://www.ncbi.nlm.nih.gov/protein/27263223?report=genbank&log$=protalign&blast_rank=1&RID=EMRYRME501S) prion-like protein [Takifugu rubripes] [emb|CAF25320.1|](http://www.ncbi.nlm.nih.gov/protein/86604469?report=genbank&log$=protalign&blast_rank=1&RID=EMRYRME501S) prion-like protein [Takifugu rubripes] Length=461

Score = 22.7 bits (47), Expect = 0.32, Method: Compositional matrix adjust.

Identities = 16/38 (42%), Positives = 19/38 (50%), Gaps = 2/38 (5%)

p300 167 TPTTPTGQQPTTPQTPQPT--SQPQPTPPNSMPPYLPR 202

TP+ P+G QP P P+ S PQP S P PR

Sbjct 47 TPSKPSGSQPNRNSNPYPSGGSYPQPGTGQSNPGGYPR 84

p300 407 NSNPQPIPG---QPGMPQGQPGLQPPTMPGQQGVHSNPAMQNMNPMQAGVQRAGLPQQQP 463

NSNP P G QPG Q PG P +Q SNP + N G P Q P

Sbjct 59 NSNPYPSGGSYPQPGTGQSNPGGYP-----RQNPASNPVGGSPNQYPGRTNPGGYPNQNP 113

p300 464 QQQLQP---PMGGMSP 476

P P GG P

Sbjct 114 AGGGYPNQNPAGGGYP 129

p300 408 SNPQPIPGQPGMPQG 422

SNP PG+ G QG

Sbjct 147 SNPNQYPGRAGTNQG 161

p300 509 GAGPGIGPGMANHNQF--QQPQGVGYPPQQQQRMQHHMQQMQQGNM 552

G P PG N + Q P G GYP Q + Q +GN

Sbjct 93 GGSPNQYPGRTNPGGYPNQNPAGGGYPNQNPAGGGYPNQNPARGNY 138

p300 451 AGVQRAGLP 459

G++ G P

Sbjct 398 VGIEEIGYP 406

p300 24 GLDDESNNQQAAATQSPGDSRRLSIQRCIQSLV 56

G DD ++ P ++ +RC++ +

Sbjct 393 GDDDTVGIEEIGY---PALVDQMKSRRCVEEYM 422

p300 613 PNSLSNQVRSPQPVPSPRPQSQPPHSSPS--PRMQPQPSPHHVSP 655

PN SN P P PQ S+P PR P +P SP

Sbjct 56 PNRNSN----PYPSGGSYPQPGTGQSNPGGYPRQNPASNPVGGSP 96

p300 349 VGISPL-KPGTVSQQALQNLLRTLRSPSSPLQQQQV 383

VGI + P V Q + + + S ++Q

Sbjct 398 VGIEEIGYPALVDQMKSRRCVEEYMAYSEQFLEKQA 433

p300 525 QQPQGVGYPPQ 535

Q Q +GY P+

Sbjct 282 QSVQSMGYKPK 292

p300 159 GLPSPTPATPTTPTGQQPT 177

G P PA + P G P

Sbjct 81 GYPRQNPA--SNPVGGSPN 97

p300 460 QQQPQQQLQP---PMGGMSPQAQ--QMNMNHNTMPSQF 492

Q P + P P GG +P + N P+Q+

Sbjct 130 NQNPARGNYPNQYPAGGSNPNQYPGRAGTNQGGYPNQY 167

**9.**[ref|NP_741772.1|](http://www.ncbi.nlm.nih.gov/protein/25150407?report=genbank&log$=protalign&blast_rank=21&RID=E9YHFVPV013) Prion-like-(Q/N-rich)-domain-bearing protein family member (pqn-65)


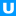

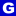

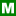


[Caenorhabditis elegans] Length=1702

[GENE ID: 180734 pqn-65](http://www.ncbi.nlm.nih.gov/sites/entrez?db=gene&cmd=search&term=180734&RID=E9YHFVPV013&log$=geneexplicitprot&blast_rank=21) | Prion-like-(Q/N-rich)-domain-bearing protein

[Caenorhabditis elegans]

Score = 20.0 bits (40), Expect = 53, Method: Compositional matrix adjust.

Identities = 13/33 (39%), Positives = 15/33 (45%), Gaps = 3/33 (9%)

p300 502 MQQQQQQGAGPGIGPGMANHNQFQQPQGVGYPP 534

M Q G G ++NQFQQP YPP

Sbjct 415 MYSNQTSSNGYYGGYEDVSNNQFQQP---DYPP 444

p300 222 TPPQTAQPPLPGPPPAAVEMAMQI 245

TPPQ PL PP E Q+

Sbjct 1542 TPPQEHSSPLTTPPIINHEYVQQV 1565

p300 69 PSCQKMKRVVQHTKGCKRKTNGGCPIC 95

PS + ++ V H + + + G PI

Sbjct 1582 PSSRPIRTGVHHREEALNEDDNGSPIL 1608

p300 619 QVRSPQPVPSPRPQSQPPHSS 639

QVRS + +PS + HS+

Sbjct 635 QVRSTKKLPSNFKEDDIRHST 655

p300 183 QPTSQPQPTPPNSMPP 198

Q T QP P+ N +PP

Sbjct 284 QSTHQPLPSVANLLPP 299

p300 536 QQQRMQHHMQQ 546

Q QRM H++Q

Sbjct 687 QSQRMDQHLEQ 697

p300 408 SNPQPIPGQPGMPQGQPGLQPPTMPGQQGVH 438

S+P I QP +PG P + P + GVH

Sbjct 1566 SSPVDIFSQPST--SEPG--PSSRPIRTGVH 1592

p300 274 PMAPMGMNPPPMTRGPSGHLEP 295

P A + ++ PP++ P EP

Sbjct 570 PFADIELSDPPLSITPQVAQEP 591

**10**.>[ref|NP_990796.1|](http://www.ncbi.nlm.nih.gov/protein/45382625?report=genbank&log$=protalign&blast_rank=3&RID=EMRYRME501S) major prion protein homolog precursor [Gallus gallus]


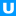

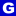

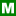


Length=267

[GENE ID: 396452 PRNP](http://www.ncbi.nlm.nih.gov/sites/entrez?db=gene&cmd=search&term=396452&RID=EMRYRME501S&log$=geneexplicitprot&blast_rank=3) | prion protein [Gallus gallus] (Over 10 PubMed links)

Score = 17.7 bits (34), Expect = 13, Method: Compositional matrix adjust.

Identities = 5/13 (38%), Positives = 7/13 (54%), Gaps = 0/13 (0%)

p300 676 HFASPDQNSMLSQ 688

HF PD+ S+

Sbjct 147 HFDRPDEYRWWSE 159

p300 414 PGQPGMPQG 422

PG PG QG

Sbjct 86 PGYPGWGQG 94

p300 344 PGLGQVGISPLKPGTVSQQ 362

PG GQ G +P G+ Q

Sbjct 89 PGWGQ-GYNPSSGGSYHNQ 106

p300 406 ANSNPQP-IPGQPGMPQGQPGL-QPPTMPGQQGVHSNPAM 443

A S+ QP P QPG P PG P P G NP

Sbjct 38 AGSHRQPSYPRQPGYPH-NPGYPHNPGYPHNPGYPHNPGY 76

p300 413 IPGQPGMPQGQPGLQPPTMPGQQGVHSNPAM----QNMNPMQAGVQRAGLPQQQPQQQLQ 468

P PG P P P G NP Q NP G P + P+ +

Sbjct 58 YPHNPGYPHNPGYPHNPGYPQNPGYPHNPGYPGWGQGYNPSSGGSYHNQKPWKPPKTNFK 117

p300 509 GAGPGIGP--GMANHNQ 523

G G G P G + HNQ

Sbjct 90 GWGQGYNPSSGGSYHNQ 106

p300 306 PPWSQGGLP 314

P W QG P

Sbjct 89 PGWGQGYNP 97

p300 527 PQGVGYP 533

PQ GYP

Sbjct 77 PQNPGYP 83

p300 156 QQQGLPSPTPATPTTPTGQQPTTPQTPQPTSQPQ-PTPPNSMPPYLPRTQAAGPVSQGKA 214

+Q G P P P P P P P PQ P P++ P Y Q P S G

Sbjct 48 RQPGYPH-NPGYPHNPG--YPHNPGYPHNPGYPQNPGYPHN-PGYPGWGQGYNPSSGGSY 103

p300 590 PMSPQQHMLPNQAQSPH 606

P P+Q P+ PH

Sbjct 44 PSYPRQPGYPHNPGYPH 60

**11**.>[gb|AAF02424.1|AF106918_1](http://www.ncbi.nlm.nih.gov/protein/6048984?report=genbank&log$=protalign&blast_rank=5&RID=EMRYRME501S) prion-like protein [Homo sapiens]


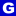


Length=176

[GENE ID: 23627 PRND](http://www.ncbi.nlm.nih.gov/sites/entrez?db=gene&cmd=search&term=23627&RID=EMRYRME501S&log$=geneexplicitprot&blast_rank=5) | prion protein 2 (dublet) [Homo sapiens]

(Over 10 PubMed links)\

Score = 17.3 bits (33), Expect = 13, Method: Composition-based stats.

Identities = 5/19 (26%), Positives = 11/19 (58%), Gaps = 0/19 (0%)

p300 301 GMQQQPPWSQGGLPQPQQL 319

G++ + W++ LP Q+

Sbjct 28 GIKHRIKWNRKALPSTAQI 46

p300 674 QGHFASPD 681

QG F PD

Sbjct 117 QGEFQKPD 124

p300 293 LEPGMGPTGMQQQP 306

LE G G QP

Sbjct 149 LERGAGLRVTMHQP 162

**12**.>[ref|NP_036541.2|](http://www.ncbi.nlm.nih.gov/protein/34335268?report=genbank&log$=protalign&blast_rank=6&RID=EMRYRME501S) prion-like protein doppel preproprotein [Homo sapiens]

[sp|Q9UKY0.2|PRND_HUMAN](http://www.ncbi.nlm.nih.gov/protein/68067934?report=genbank&log$=protalign&blast_rank=6&RID=EMRYRME501S) RecName: Full=Prion-like protein doppel; AltName: Full=PrPLP;

AltName: Full=Prion protein 2; Flags: Precursor Length=176

[GENE ID: 23627 PRND](http://www.ncbi.nlm.nih.gov/sites/entrez?db=gene&cmd=search&term=23627&RID=EMRYRME501S&log$=geneexplicitprot&blast_rank=6) | prion protein 2 (dublet) [Homo sapiens]

(Over 10 PubMed links)

Score = 17.3 bits (33), Expect = 13, Method: Composition-based stats.

Identities = 5/19 (26%), Positives = 11/19 (58%), Gaps = 0/19 (0%)

p300 301 GMQQQPPWSQGGLPQPQQL 319

G++ + W++ LP Q+

Sbjct 28 GIKHRIKWNRKALPSTAQI 46

p300 293 LEPGMGPTGMQQQP 306

LE G G QP

Sbjct 149 LERGAGLRVTMHQP 162

**13.>**[ref|NP_501888.2|](http://www.ncbi.nlm.nih.gov/protein/71992530?report=genbank&log$=protalign&blast_rank=7&RID=E9YHFVPV013) Prion-like-(Q/N-rich)-domain-bearing protein family member (pqn-55)


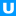

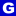

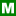


[Caenorhabditis elegans] Length=1717

[GENE ID: 177915 pqn-55](http://www.ncbi.nlm.nih.gov/sites/entrez?db=gene&cmd=search&term=177915&RID=E9YHFVPV013&log$=geneexplicitprot&blast_rank=7) | Prion-like-(Q/N-rich)-domain-bearing protein

[Caenorhabditis elegans] (10 or fewer PubMed links)

p300 76 RVVQHTKGCKRKTNGGCPICKQLIALCCY 104

R+V+ T C +++ CK ++AL Y

Sbjct 241 RIVECTCECSNRSSW----CKHVVALSIY 265

p300 169 TTPTGQQPTTPQTPQPTSQPQPTPPNSMPPYLPRTQAAG 207

+T +GQ+ +TPQ P+ +S+ + AA

Sbjct 664 STESGQESDVQKTPQRVEIPRKKKVDSVEMDVNEVLAAA 702

p300 89 NGGCPICKQLIALCC 103

NG PI +L CC

Sbjct 1624 NGFQPITGELYERCC 1638

p300 44 RRLSIQRCI 52

RRL I+ C+

Sbjct 354 RRLLIKYCV 362

p300 622 SPQPVPSPRPQSQPPHSSPS 641

S +PVP+ QS PP +SP+

Sbjct 909 SIRPVPA---QSVPPTASPA 92

p300 411 QPIPGQPGMPQGQPGLQ 427

+P+P Q P P LQ

Sbjct 911 RPVPAQSVPPTASPALQ 927

p300 184 PTSQPQPTPPNSMPP 198

P++ +P P S+PP

Sbjct 906 PSTSIRPVPAQSVPP 920

p300 46 LSIQRCIQSLVHACQC--RNANCSLPSCQKMKRVVQHTKGCKRKT 88

+ + RC + + C+C R++ C + R+ + + ++T

Sbjct 235 VKVDRC-RIVECTCECSNRSSWCKHVVALSIYRIYERSNIKFKET 278

p300 227 AQPPLPGPPPAAVEMA-MQIQRAAETQRQM 255

AQ P+P P +A + Q AE QR +

Sbjct 1040 AQEPVPESAPNRCLIADYEAQIEAELQRMI 1069

p300 441 PAMQNMNPMQAGVQRAGLP 459

PA+Q N + R G+P

Sbjct 573 PAIQMCNYLNEKDIRFGVP 591

**14**.>[ref|NP_496262.2|](http://www.ncbi.nlm.nih.gov/protein/71991056?report=genbank&log$=protalign&blast_rank=9&RID=E9YHFVPV013) Prion-like-(Q/N-rich)-domain-bearing protein family member (pqn-47)

[Caenorhabditis elegans] Length=931

[GENE ID: 174614 pqn-47](http://www.ncbi.nlm.nih.gov/sites/entrez?db=gene&cmd=search&term=174614&RID=E9YHFVPV013&log$=geneexplicitprot&blast_rank=9) | Prion-like-(Q/N-rich)-domain-bearing protein

[Caenorhabditis elegans] (10 or fewer PubMed links)

Score = 22.3 bits (46), Expect = 12, Method: Compositional matrix adjust.

Identities = 22/95 (23%), Positives = 42/95 (44%), Gaps = 8/95 (8%)

p300 15 DHKMEKLGLGLDDESNNQQAAATQSP---GDSRRLSIQRCIQSLVHACQCRNANCSLPSC 71

D K+ ++ L++ + ++ A++ + GD++ LS RC SL NA S P

Sbjct 583 DEKVAEISRRLNEYAVRKKLASSMASNLNGDNKSLSYSRC--SLTSTAT--NAT-SQPKR 637

p300 72 QKMKRVVQHTKGCKRKTNGGCPICKQLIALCCYHA 106

+ R ++ + C + + G + I C A

Sbjct 638 SRKHRAIKQAQSCGSRLSQGTVVTLVSIMAACLLA 672

p300 431 MPGQQGVHSNPAMQNMNP 448

MP Q +H+ MQNMN

Sbjct 146 MPQQSSIHAQ--MQNMNA 161

p300 175 QPTTPQTPQPTSQPQPTPPNSMPPYLP 201

+ TP T + +P N MP + P

Sbjct 693 ETNTPSTKGELANLVISPANFMPSFQP 719

p300 125 KLRQQQLQHRLQQAQMLRRRMASMQRTGVVGQQ 157

KLR +++ + A + RTG++ Q+

Sbjct 505 KLRVVDYRYKPEVADIWGLDEQQRHRTGLIAQE 537

p300 155 GQQQGLPSPTPATP 168

G G+PSP P

Sbjct 192 GADSGMPSPVLEMP 205

**15**.>[ref|NP_495966.2|](http://www.ncbi.nlm.nih.gov/protein/115533990?report=genbank&log$=protalign&blast_rank=10&RID=E9YHFVPV013) Prion-like-(Q/N-rich)-domain-bearing protein family member (pqn-95)


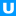

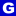

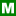


[Caenorhabditis elegans] Length=373

[GENE ID: 174464 pqn-95](http://www.ncbi.nlm.nih.gov/sites/entrez?db=gene&cmd=search&term=174464&RID=E9YHFVPV013&log$=geneexplicitprot&blast_rank=10) | Prion-like-(Q/N-rich)-domain-bearing protein

[Caenorhabditis elegans] (10 or fewer PubMed links)

Score = 21.9 bits (45), Expect = 12, Method: Compositional matrix adjust.

Identities = 10/20 (50%), Positives = 11/20 (55%), Gaps = 0/20 (0%)

p300 608 QGQQIPNSLSNQVRSPQPVP 627

QG NS SNQ +S P P

Sbjct 250 QGDSCSNSCSNQCQSACPTP 269

p300 162 SPTPATPTTPTGQQPTTPQTPQPTSQPQPTPPNS 195

S + +TP Q + Q QPT QPQ P S

Sbjct 201 SSSCSTPICIQSCQSSCQQACQPTCQPQCMPSCS 234

p300 10 NTKNHDHKMEKLGLGLDDESNNQQAAATQSPGDSRRLSIQRCIQSLVHACQ 60

N N + + NNQ +++ +P + IQ C S ACQ

Sbjct 177 NNNNQQIIVVQQDNSCSSNCNNQCSSSCSTP-----ICIQSCQSSCQQACQ 222

WS P Q SG R + ++ Q G

Sbjct 30 WSTMSGSGPNQAMSGQRRNSWNNLVQVG 57

>[ref|NP_500906.2|](http://www.ncbi.nlm.nih.gov/protein/71984800?report=genbank&log$=protalign&blast_rank=12&RID=E9YHFVPV013) Prion-like-(Q/N-rich)-domain-bearing protein family member (pqn-22)


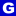

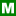


[Caenorhabditis elegans] Length=925

[GENE ID: 177369 pqn-22](http://www.ncbi.nlm.nih.gov/sites/entrez?db=gene&cmd=search&term=177369&RID=E9YHFVPV013&log$=geneexplicitprot&blast_rank=12) | Prion-like-(Q/N-rich)-domain-bearing protein

[Caenorhabditis elegans] (10 or fewer PubMed links)

Score = 21.6 bits (44), Expect = 19, Method: Compositional matrix adjust.

Identities = 12/41 (29%), Positives = 20/41 (49%), Gaps = 0/41 (0%)

p300 485 HNTMPSQFRDILRRQQMMQQQQQQGAGPG 513

HN +RD +++ Q A PG

Sbjct 852 HNNTEGGYRDANGHDVSYKRETQTSADPG 880

p300 445 NMNPMQAGVQRAGLPQQQPQQQLQPPMGGMS 475

N +R Q PQQ+LQ P GG S

Sbjct 734 NQESYTTHTERNNSSQGFPQQRLQ-PSGGYS 763

p300 348 QVGISPLKPGTVSQQALQNLL 368

+V +PL+PG +S+

Sbjct 892 RVVETPLEPGVISRHVTTKYY 912

p300 70 SCQKMKRVVQHTKGCKRKTNGG 91

S QK++ V+H K T+ G

Sbjct 274 SNQKIRHEVRHIDQTKFGTSFG 295

p300 44 RRLSIQRCIQSLVHACQCRNANCSLPSCQKMKRVVQHTKGCKRKTNG 90

+ LS +C+ + + + N SLP + +T+G R NG

Sbjct 821 KDLSNAQCVDDVFN--KKTEMNESLP-VGSVFNTHNNTEGGYRDANG 864

p300 221 PTPPQTAQPPLPGPPPA 237

P P TA P P PA

Sbjct 102 PADPHTAYRPASSPAPA 118

p300 601 QAQSPHLQGQQIPNSLSNQVRSPQPVPSPRPQSQPPHSSPSPRMQPQPSPHHVSPQTSSP 660

QA++P + Q PN + S P PH++ P P P+ H V + +

Sbjct 72 QAEAPRVPYQSEPNWARTAREKSERARSKTPAD--PHTAYRPASSPAPANHPVHAENARE 129

p300 661 H 661

H

Sbjct 130 H 130

Sbjct 35 RKPTPSPSQVTEARVAMTRP 54
